# Breaking the siege of hypoxia and lactate: carrier-free flexible dual-enzyme protein vesicle to ignite photodynamic-immune storm in head and neck squamous cell carcinoma

**DOI:** 10.1016/j.mtbio.2025.102082

**Published:** 2025-07-12

**Authors:** Yan Zhou, Xiaoquan Xu, Ziyue Zu, Shangyu Lu, Wei Lu, Xi Luo, Nan Zhong, Yang Liu, Zhaogang Teng, Shouju Wang, Feiyun Wu

**Affiliations:** a Laboratory of Molecular Imaging, Department of Radiology, The First Affiliated Hospital with Nanjing Medical University, PR China; b Key Laboratory for Organic Electronics and Information Displays and Jiangsu Key Laboratory for Biosensors, Institute of Advanced Materials, Jiangsu National Synergetic Innovation Centre for Advanced Materials, Nanjing University of Posts and Telecommunications, PR China

**Keywords:** Head and neck squamous cell carcinoma, Hypoxia, Lactic acid, Protein vesicle, Photodynamic therapy

## Abstract

Photodynamic therapy (PDT) efficacy in head and neck squamous cell carcinoma (HNSCC) is hindered by hypoxia and lactate accumulation in tumor microenvironment (TME), which suppress reactive oxygen species (ROS) generation and immune activation. To address this, we developed a carrier-free flexible dual-enzyme protein vesicle by covalently conjugating human serum albumin (HSA), catalase (CAT), and lactate oxidase (LOX) onto a mesoporous silica template, followed by template etching. Subsequently, the near-infrared photosensitizer IR808 was incorporated to obtain the final nanoparticle (HSA/CAT/LOX@IR808). This system employs a self-sustaining enzymatic cascade: CAT alleviates hypoxia by converting tumor-derived H_2_O_2_ into oxygen, while LOX depletes lactate and generates additional H_2_O_2_ to fuel CAT activity, synergistically amplifying ROS production. The flexible structure enhances tumor penetration of the nanoparticle, and the incorporation of HSA further enhances endothelial cell transport. *In vitro and in vivo* studies demonstrated the excellent biocompatibility, tumor-targeting capability, and TME remodeling effects of the vesicle. The vesicle triggered robust immune activation, characterized by M1 macrophage polarization, dendritic cell maturation, and CD8^+^ T cell infiltration. Ultimately, HSA/CAT/LOX@IR808-mediated PDT led to marked tumor inhibition, prolonged survival, and reduced metastasis. These findings underscore the potential of our protein vesicle as an effective and safe therapeutic approach for HNSCC.

## Graphical abstract

**Figure:**
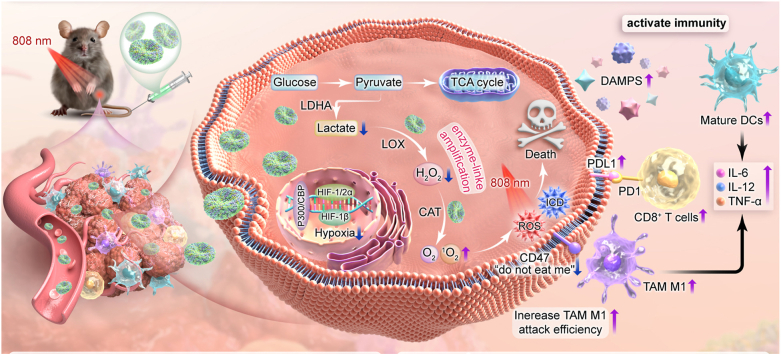

## 1 Introduction

Head and neck cancer (HNC) ranks among the six most common malignancies worldwide, with nearly 900,000 new cases and 500,000 deaths annually [[1], [2], [3]]. Head and neck squamous cell carcinoma (HNSCC) accounts for approximately 90 % of HNC, with most patients diagnosed at an advanced stage. Its high recurrence rate and resistance to standard therapies, compounded by the complex anatomy of the region, pose significant clinical challenges [[1], [2], [3], [4], [5]]. Photodynamic therapy (PDT) offers a promising, minimally invasive alternative, and several PDT-based therapies have already been approved for clinical use [[6], [7], [8]]. Beyond its precision, PDT also triggers immune activation through the induction of immunogenic cell death (ICD), fostering long-term immune memory, and improving immune response rates [[9], [10], [11], [12]]. Nevertheless, the effectiveness of PDT in HNSCC is often hindered by the complex and hostile tumor microenvironment (TME), which plays a critical role in promoting tumor progression and immune suppression. Therefore, strategies aimed at improving the TME are crucial for optimizing the effectiveness of PDT.

Hypoxia is one of the most critical TME factors that hampers the efficacy of PDT and has been extensively studied [[13], [14], [15], [16], [17], [18]]. Numerous studies have explored strategies to overcome hypoxia, including oxygen-releasing nanocarriers and oxygen-generating agents. While promising, the therapeutic efficiency still require improvement, possibly because they address only a single aspect of the TME. Under hypoxic conditions, tumor cells often switch to anaerobic glycolysis, a phenomenon referred to as the Warburg effect, leading to a significant accumulation of lactate. Elevated lactate levels further aggravate hypoxia by stabilizing hypoxia-inducible factor-1α (HIF-1α) and impairing oxygen diffusion. Moreover, lactate plays a direct role in promoting tumor progression by facilitating immune evasion, angiogenesis, and extracellular matrix remodeling [[19], [20], [21], [22], [23]]. This establishes a self-reinforcing loop between hypoxia and lactate accumulation, creating a hostile TME that fuels tumor aggressiveness and further compromises PDT outcomes. Therefore, dual targeting of both hypoxia and lactate represents a more comprehensive and rational approach to reprogramming the TME and overcoming PDT resistance.

To address this critical issue, emerging nanotherapeutic approaches have attempted to tackle this dual regulatory challenge through co-delivery systems [[23], [24], [25], [26], [27], [28], [29]]. As documented in some studies, certain co-delivery methods have been developed to simultaneously administer oxygen-generating and lactate-metabolizing agents. Complementarily, some investigators have engineered cascade nanoenzymes to enhance therapeutic efficacy [[26], [27], [28], [29], [30], [31]]. For example, Liu et al. [29] functionalized iridium metallenes with catalase (CAT)-like activity and conjugated them to lactate oxidase (LOX), thereby achieving a cascade amplification effect. Despite these advancements, current nanocarriers predominantly rely on rigid inorganic frameworks, such as silica nanoparticles, manganese-based nanomaterials, or metal-organic frameworks (MOFs) [[26], [27], [28], [29], [30], [31]]. The drug loading capacity of these systems is closely related to their structural properties, which may vary depending on the specific carrier design. Furthermore, the potential toxicity of metal ions may raise concerns regarding biosafety. Additionally, researches have shown that the inflexibility of rigid nanomaterials restrict their tumor penetration ability [[32], [33], [34]]. They generally demonstrate lower efficiency in cellular internalization, drug delivery, and tumor-targeted accumulation when compared to their flexible counterparts.

**Scheme 1:**
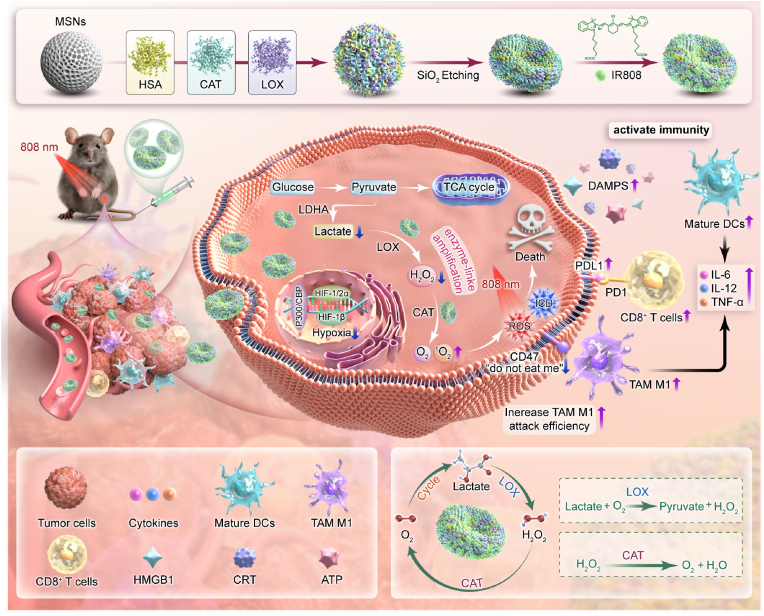
An overview of how the flexible dual-enzyme protein vesicle alleviates the hypoxic and lactate-rich TME, enhancing ROS generation through cascade reactions. This leads to improved PDT efficacy, ICD induction, and immune system activation, offering a promising new approach to improving treatment outcomes in HNSCC.

Herein, we formulated a carrier-free flexible dual-enzyme protein vesicle through covalent conjugation of human serum albumin (HSA), CAT, and LOX onto sacrificial mesoporous silica templates, followed by template removal via chemical etching. The near-infrared photosensitizer IR808 was subsequently added, yielding the functional nanoparticle system (HSA/CAT/LOX@IR808). In this system, CAT converts hydrogen peroxide (H_2_O_2_) into oxygen, relieving hypoxia, while LOX catalyzes the oxidation of lactate, reducing lactate accumulation and producing additional H_2_O_2_ as a substrate for CAT. This creates a synergistic amplification loop, where in each enzyme enhances the other's activity, significantly improving both the hypoxic and lactate-rich TME. The presence of IR808, activated by NIR light, further amplifies reactive oxygen species (ROS) generation. The carrier-free flexible architecture enhances tumor targeting efficiency, while HSA facilitates endothelial transport [[34], [35], [36]]. The integration of carrier-free flexible design and dual-enzyme synergy effectively modulates the hypoxic and lactate-rich TME, consequently enhancing PDT efficacy and immune activation in HNSCC treatment (Scheme 1).

## 2 Materials and methods

### 2.1 Synthesis of the protein vesicle

Initially, 0.32 g of Cetyltrimethylammonium bromide (CTAB) was dissolved in a solution of 60 mL ethanol, 150 mL water, and 1 mL NH_3_·H_2_O. The mixture was stirred at 500 rpm in a 35 °C water bath for 1 h. Next, 1 mL tetraethoxysilane (TEOS) was added, and the mixture was stirred for an additional 3 h. The product was washed with water and ethanol, then dispersed in 20 mL ethanol. To remove CTAB, the product was treated three times with 200 mL ethanol containing 400 μL concentrated HCl, stirred at 60 °C, and then washed with ethanol and water. The CTAB-free mesoporous silica nanoparticles (MSNs) were dispersed in 25 mL deionized water.

MSNs (10 mL, 4 mg/mL) were mixed with 2 mL polyethylenimine (PEI) (10 mg/mL) and 8 mL water, shaken, centrifuged, and washed. The resulting MSNs-PEI were ultrasonically dispersed in 15 mL water with 5 mL glutaraldehyde (GA) and agitated for 12 h. After washing, the MSNs-PEI-GA solution was obtained. To activate CAT, 3 mg of CAT was dissolved in 8 mL of water, followed by the addition of 1 mL of EDC (3 mg/mL) and 1 mL of NHS (3 mg/mL). The solution was shaken at room temperature for 8 h. Similarly, 2 mg of LOX was dissolved in 2 mL of water, then mixed with 2 mL of EDC (2 mg/mL) and 2 mL of NHS (2 mg/mL), and shaken for 8 h to complete activation. The obtained MSNs-PEI-GA were dispersed in 15 mL of water, followed by sequential addition of 5 mL of HSA (2 mg/mL, totaling 10 mg) and the above-activated CAT and LOX. The mixture was gently shaken for 12 h. Subsequently, 5 mL of 5 % (v/v) hydrofluoric acid (HF) solution was added to the suspension, followed by gentle mixing for 1 min [34]. The mixture was then immediately centrifuged at 15,000 rpm for 3 min to separate the etched product. Finally, HSA/CAT/LOX was obtained. IR808 was activated by dissolving 2 mg IR808 in 1 mL N,N-dimethylformamide (DMF), mixing with 2 mL hydrochloride (EDC) (1 mg/mL) and 2 mL N-hydroxysulfosuccinimide (NHS) (1 mg/mL), and shaking for 8 h in the dark. The activated IR808 was added to the HSA/CAT/LOX and shaken for 12 h to yield the final HSA/CAT/LOX@IR808 product.

The encapsulation efficiency (EE) and loading capacity (LC) of HSA, CAT, and LOX in the nanovesicles were quantified using ELISA kits specific for each protein. The IR808 content was quantified by UV–vis spectroscopy based on absorbance at 808 nm. Based on these quantification results, the final loading ratios of HSA, CAT, LOX, and IR808 in the HSA/CAT/LOX@IR808 were calculated.

### 2.2 Characterization

High-resolution transmission electron microscopy (TEM) and elemental mapping, including Energy Dispersive X-ray Spectroscopy (EDS), were conducted on the samples to gather structural and elemental data. The hydrodynamic size and Zeta potential of the prepared samples were measured using a NanoBrook ZetaPALS instrument. Atomic force microscope (AFM) was used to evaluate the flexibility of the materials by measuring Young's modulus. Fourier transform infrared (FT-IR) spectra were obtained utilizing the potassium bromide pellet method with an In10IZ10 spectrometer. UV–vis spectra were also recorded. Fluorescence (FL) spectra were analyzed. The dispersion and stability of HSA/CAT/LOX@IR808 in PBS (pH 7.4) were assessed for a 7-day period. The stability of the materials was tested over 30 days by measuring the hydrodynamic size and Zeta potential every 5 days, with TEM used to observe morphological changes.

### 2.3 Enzyme activity assessment

To observe the catalytic ability of CAT, 1 mL of HSA (1 mg/mL), 1 mL of CAT (1 mg/mL), 1 mL of LOX (1 mg/mL), 1 mL of IR808 (50 μg/mL), and 1 mL of HSA/CAT/LOX@IR808 (with CAT concentration calculated at 1 mg/mL) were separately added into 5 mL of PBS solution with or without H_2_O_2_ addition. These mixtures were placed in a water bath at 37 °C for 30 min incubation, and oxygen bubble production was visually observed as an indication of catalysis.

For quantitative assessment of the enzyme activity, Michaelis-Menten kinetics was applied to measure changes in enzyme function before and after HF etching. The catalytic activity of CAT was assessed by measuring the consumption of H_2_O_2_, and LOX activity was determined by monitoring the production of H_2_O_2_. H_2_O_2_ consumption and production were quantified by monitoring the changes in absorbance at 240 nm, using a UV–vis spectra.

### 2.4 Synergistic enzyme cascade amplification assessment

To evaluate the synergistic enzyme cascade amplification effect, as well as the effect of different enzyme ratios, changes in lactate concentration, pH, and H_2_O_2_ levels were monitored over time after adding 10 mM lactate to the different groups: PBS, LOX, HSA, HSA/CAT, HSA/LOX, and HSA/CAT/LOX. The enzyme ratios for the HSA/CAT/LOX formulations were adjusted to 4:1, 3:1, 2:1, 1:1, and 1:2 (CAT:LOX). Each formulation was prepared in PBS to maintain the same overall material concentration (1 mg/mL) across groups. Samples were collected at 0, 1, 3, 5, and 10 min, and lactate levels were quantified using a lactate assay kit, pH was measured continuously with a pH test strips, and H_2_O_2_ levels were assessed using a H_2_O_2_ assay kit. This setup allowed for the comparative analysis of individual and combined effects of the enzymes.

### 2.5 Enzyme-linked amplification for ROS generation

For light-induced ROS production, different materials were incubated in PBS and irradiated with an 808 nm laser. To assess substrate-induced ROS production, lactate (10 mM) and H_2_O_2_ (100 μM) were added to the materials. Various loading ratios of CAT and LOX (4:1, 3:1, 2:1, 1:1, 1:2) were tested to determine the optimal combination for maximum ROS generation. Singlet oxygen (^1^O_2_) generation was assessed using Singlet Oxygen Sensor Green (SOSG, 50 μM), a selective fluorescent probe for ^1^O_2_. Briefly, 100 μL of each nanomaterial formulation was mixed with 50 μL of SOSG working solution in a quartz cuvette and irradiated with an 808 nm laser at a power density of 0.5 W/cm^2^ for 0, 1, 3, 5, and 10 min.

### 2.6 Cell lines

SCC7, HN6, FaDu, RAW264.7 and 3T3 cell lines were obtained from Procell Life Science & Technology Company. Cells were cultured in RPMI 1640 or DMEM, supplemented with 10 % FBS and 1 % PS solution, at 37 °C with 5 % CO_2_.

### 2.7 Cellular uptake assessment

To assess the cellular uptake of different nanomaterials, SCC7 cells (1 × 10^5^) were seeded in a 12-well plate and treated with MSN/CAT/LOX@IR808, CAT/LOX@IR808, and HSA/CAT/LOX@IR808 for 1, 3, and 6 h. Confocal laser scanning microscopy was employed to image the FL and intracellular localization of the nanomaterials. Additionally, biological electron microscopy was used to analyze nanoparticle uptake and intracellular distribution.

### 2.8 Detection of intracellular lactate and H_2_O_2_ levels

SCC7 cells were seeded in 6-well plates and incubated overnight. Cells were then treated with different nanomaterials for 6 h, followed by 3 min irradiation (0.5 W/cm^2^). After 6, 12, and 24 h of incubation, the cells were lysed with RIPA buffer, and the lysates were collected. Following centrifugation, the supernatants were analyzed.

For intracellular lactate detection, the CheKine™ lactate assay kit was used. Supernatants were mixed with assay buffer, lactate dehydrogenase, cofactor, WST-8, and enhancer according to the kit instructions. The absorbance was measured at 450 nm, and lactate levels were determined using a standard curve.

Intracellular H_2_O_2_ levels were measured using the CheKine™ micro H_2_O_2_ assay kit. The supernatants were mixed with assay reagents as per the kit instructions, and the reaction was incubated at room temperature. Absorbance was measured at 580 nm using a microplate reader, with H_2_O_2_ levels calculated from a standard curve.

### 2.9 HIF-1α expression in SCC7 cells

To quantify changes in HIF-1α expression in SCC7 cells after incubation with different materials, both western blotting (WB) and quantitative real-time PCR (qPCR) analyses were performed. SCC7 cells were seeded in 6-well plates and allowed to adhere overnight. The cells were then incubated with different materials for 6 h. After a 3-min irradiation laser (0.5 W/cm^2^), the cells were incubated for an additional 36 h. For WB, cells were lysed and protein concentrations measured. Equal amounts of protein were separated by SDS-PAGE, transferred to membranes, and probed with HIF-1α and GAPDH antibodies, followed by ECL detection. For qPCR, RNA was extracted, converted to cDNA, and analyzed with specific primers for HIF-1α. The forward primer is 5′-CACTCCTAACTTTTCCCAGCCTA-3′, and the reverse primer is 5′-TGGATTCTTTGCCTCTGTGTCT-3′, with a product length of 130 bp. HIF-1α expression was calculated relative to GAPDH.

### 2.10 Intracellular ROS generation assay

To assess the enzyme-linked amplification of ROS generation, SCC7, HN6, and FaDu cells were treated with various formulations, including PBS, IR808, HSA@IR808, HSA/CAT@IR808, and HSA/CAT/LOX@IR808, followed by 808 nm laser irradiation for 3 min (0.5 W/cm^2^). ROS generation was measured using DCFH-DA with FL microscope and flow cytometry. Additionally, the effect of LOX and CAT inhibitors (FOP, 3-AT, and combined) was tested to verify the role of enzyme-linked amplification in SCC7 cells. These analyses allowed for a detailed evaluation of ROS production and amplification under laser treatment with and without enzyme inhibition.

### 2.11 Cell cytotoxicity assay

To evaluate the cytotoxic effects of various treatments, 808 nm irradiation durations, and IR808 concentrations on SCC7, HN6, and FaDu cells, the CCK-8 assay was employed. Cells were seeded in 96-well plates and incubated overnight. Following treatment with different materials and IR808 concentrations (0–20 μg/mL IR808) for 6 h, the cells were exposed to an 808 nm laser at intervals of 0, 1, 3, and 5 min (0.5 W/cm^2^) and incubated for an additional 3 h. Cell viability was determined by adding 10 μL of CCK-8 solution to each well, followed by a 2-h incubation at 37 °C. Absorbance at 450 nm was measured to quantify cytotoxic effects across different treatment groups. The half-maximal inhibitory concentration (IC_50_) values were calculated for each cell line.

### 2.12 Live/dead viability assay

To further assess cell viability after treatment, a live/dead viability assay was performed. Calcein-AM was used to stain live treated SCC7 cells, while PI was used for dead cells. The cells were treated with IR808 at a concentration of 10 μg/mL and exposed to an 808 nm laser at intervals of 3 min (0.5 W/cm^2^). The live/dead cell counts were quantified, providing a visual confirmation of the cytotoxic effects.

### 2.13 Apoptosis detection

To detect apoptosis in treated SCC7 cells, flow cytometry was used. Cells were harvested, washed in PBS, and stained with Annexin V-FITC and PI, which permitted the classification of cells into live, early apoptotic, late apoptotic, and necrotic categories based on their staining profiles.

### 2.14 Cell proliferation assay

To assess the long-term effects of treatments on cell proliferation, a clone formation assay was performed. SCC7 cells were seeded at a low density (500 cells per well) in 6-well plates and allowed to adhere overnight. Following treatment with different materials (2 μg/mL IR808) and subsequent irradiation with an 808 nm laser (0.5 W/cm^2^) for 3 min as required, the cells were incubated for 12 days to allow colony formation. At the conclusion of the incubation, colonies were fixed using 4 % paraformaldehyde for 15 min and stained with 0.5 % crystal violet for 30 min. A microscope was used to count colonies with at least 50 cells.

### 2.15 Cell migration and invasion ability

To evaluate cell migration and invasion abilities, scratch tests and transwell migration and invasion assays were conducted.

SCC7 cells were seeded in 6-well plates to reach nearly 100 % confluence overnight. A scratch was made in the cell monolayer using a sterile pipette tip. Cells were washed with PBS and treated with various materials, including IR808 at a concentration of 2 μg/mL. If necessary, the cells were irradiated with an 808 nm laser (0.5 W/cm^2^) for 3 min. Images of the scratch were taken at 0, 24, and 48 h, and the percentage of wound closure was calculated.

SCC7 cells were resuspended in serum-free medium and added to the upper chamber of transwell inserts (8 μm pore size). The lower chamber contained medium with 10 % FBS. Treatments included different materials (2 μg/mL IR808), with or without laser irradiation (0.5 W/cm^2^). After 24 h, migrated cells on the lower side of the membrane were fixed, stained, and counted. For the migration assay, the transwell inserts were pre-coated with matrigel. Cells were added to the upper chambers, and medium with 10 % FBS was added to the lower chambers. After treatment and 24-h incubation, invaded cells on the lower side of the membrane were fixed, stained, and counted.

### 2.16 ICD detection

To assess ICD, SCC7 cells were seeded in 12-well plates and incubated overnight to allow for proper adhesion. The cells were then treated with different nanomaterials and incubated for 6 h. Following the incubation, the cells were exposed to IR808 light at a power density of 0.5 W/cm^2^ for 3 min to induce PDT.

After irradiation, the cells were incubated for an additional 24 h. FL staining was then performed to detect the expression of nuclear HMGB1. Cells were fixed, permeabilized, and blocked, then incubated with primary HMGB1 antibodies overnight. After washing, cells were treated with fluorescent secondary antibodies and DAPI for nucleus staining. Additionally, the supernatants from the culture were gathered, and the ATP content in the supernatants was measured using an ATP ELISA detection kit.

### 2.17 CD47 expression in SCC7 cells and macrophage phagocytosis assay

To assess CD47 expression in SCC7 cells, flow cytometry was used. The cells were gathered, washed with PBS, and then incubated with anti-CD47 antibody for half an hour in the dark. CD47 expression was quantified by measuring FL intensity with a flow cytometer.

To further evaluate macrophage phagocytosis, SCC7 cells were labeled with Cell-Tracker Green CMTPX, and RAW264.7 macrophages were labeled with Cell-Tracker Red CMTPX. Both cell types were then digested and co-incubated in serum-free medium for 6 h. Post-incubation, cells were washed with PBS and analyzed using a FL microscopy to observe and quantify the phagocytic interaction between SCC7 cells and RAW264.7 macrophages.

### 2.18 Macrophage polarization and DC maturation *in vitro*

Primary cells were extracted from mouse bone marrow and cultured in DMEM for one week. M-CSF was used to induce differentiation into bone marrow-derived macrophages (BMDMs), while IL-4 and GM-CSF were used to differentiate cells into bone marrow-derived dendritic cells (BMDCs). Separately, SCC7 Cells were placed in 6-well plates and exposed to various nanomaterial irradiated with IR808 (0.5 W/cm^2^) for 3 min, followed by a 24-h incubation. Supernatants from these SCC7 cells were then collected and added to wells containing the differentiated BMDMs or BMDCs.

After an additional 48-h incubation, BMDMs and BMDCs were harvested, resuspended in PBS, and stained with appropriate antibodies (7-AAD viability staining solution, anti-CD45-Percp, anti-CD11b-BV421, F4/80-APC, CD86-PE for BMDMs; and 7-AAD viability staining solution, anti-CD45-Percp, anti-CD11c-BV421, CD86-PE, CD80-FITC for BMDCs). These cells were then assessed using flow cytometry. Additionally, supernatants were analyzed for TNF-α, IL-12, and IL-6 levels using the ELISA method.

### 2.19 Flow cytometric analysis of PD-L1 expression after PDT treatment

Cells were seeded in 6-well plates, treated, and incubated overnight. The following day, cells were irradiated with an 808 nm laser (0.5 W/cm^2^) for 3 min and incubated for 36 h. After washing, cells were centrifuged, resuspended in PBS, and stained with PD-L1-specific fluorescent antibodies for 30 min. PD-L1 expression was analyzed by flow cytometry to assess immune responses.

### 2.20 Animals and tumor models

All animal experiments were approved by the Institutional Animal Care and Use Committee (IACUC) (Approval No. 2310061) and performed in accordance with relevant guidelines and regulations. To establish the tumor model, female nude mice and C3H/He mice (6 weeks old) were subcutaneously injected in the right flank with a suspension of 1 × 10^6^ SCC7 cells in 100 μL of saline, respectively.

### *2.21* *In vivo* FL imaging and biodistribution

100 μL of MSN/CAT/LOX@IR808, CAT/LOX@IR808, and HSA/CAT/LOX@IR808 was injected into the tail vein of nude mice with established subcutaneous tumors. FL imaging was performed at 0, 1, 3, 6, 12, 24, 48, and 96 h post-injection. The mice were anesthetized with isoflurane before imaging using a multi-mode live small animal imager. For biodistribution analysis, at each of the designated time points, the mice were sacrificed, and the heart, liver, spleen, lungs, kidneys, and tumor were excised for vivo FL imaging. The samples were excited at 780 nm using a xenon lamp with a bandpass filter, and the FL was collected at 850 nm using another bandpass filter. To assess blood pharmacokinetics, 20 μL of blood was collected from the tail vein of each mouse at selected time points into heparinized tubes. Plasma was separated by centrifugation and the FL intensity was measured. A two-compartment model for pharmacokinetic analysis was built using Drug and Statistics (DAS) software.

### 2.22 Intratumoral oxygenation monitoring by photoacoustic imaging

After intravenous tail vein injection of the protein vesicles, tumor-bearing mice were subjected to PDT with 808 nm laser irradiation. To assess the real-time changes in tumor oxygenation, mice were imaged using the LOIS-3D Plus system, a non-invasive photoacoustic imaging technique. Oxygenation levels within the tumor region were quantified by analyzing the photoacoustic signals corresponding to oxygenated hemoglobin.

### 2.23 Intratumoral lactate quantification

Tumor samples were immediately harvested, immersed in cold PBS, and homogenized using a homogenizer. The homogenates were centrifuged at 20,000×*g* for 15 min at 4 °C, and the supernatant was collected. To detect lactate concentrations, 50 μL of the tumor supernatant was mixed with 50 μL of the reaction solution containing LOX, horseradish peroxidase, and Amplex Red reagent in a black 96-well plate. After incubation at 37 °C for 30 min, FL signals were measured at excitation 571 nm and emission 585 nm using a microplate reader. To measure H_2_O_2_ abundance in tumor tissues, the supernatant was diluted 10-fold with PBS. Then, 20 μL of the diluted supernatant was mixed with 80 μL of Amplex Red solution containing 50 μM Amplex Red and 0.1 U/mL horseradish peroxidase in a black 96-well plate. After incubation at 37 °C for 30 min, FL signals were measured at excitation 530 nm and emission 590 nm with a plate reader.

### 2.24 Intratumoral HIF-*1α* and CRT detection

FL staining was conducted on tumor sections to detect HIF-1α and CRT levels. Tumor sections were collected, fixed, and embedded in paraffin. After deparaffinization and antigen retrieval, sections were permeabilized and blocked. Primary antibodies against HIF-1α or CRT were incubated overnight at 4 °C, followed by incubation with fluorescent secondary antibodies and DAPI for nuclear staining. Sections were mounted and imaged using FL microscopy. FL intensity was measured to evaluate HIF-1α and CRT expression.

### 2.25 Intratumoral ROS generation detection

To detect ROS using the DCFH-DA fluorescent probe, the probe was diluted with PBS at a ratio of 1:100. A volume of 100 μL of the diluted probe was injected into the tail vein of the mice. Twelve hours post-injection, the nanomaterials were administered, followed by IR808 irradiation (0.5 W/cm^2^) for 5 min. The tumor tissues were protected from light. Tumor sections were then prepared and stained with DAPI. The sections were scanned, and the FL intensity was quantified within the tumor sections.

### 2.26 Anti-tumor therapy *in vivo*

When the tumor volume reached approximately 100 mm^3^, C3H/He mice were randomly divided into different treatment groups. Mice received intravenous nanomaterials injections via the tail vein (1 mg/kg), followed by laser irradiation (0.5 W/cm^2^ for 5 min) 12 h later. The 1 mg/kg dose was chosen based on prior dose-escalation experiments, which indicated optimal therapeutic efficacy and safety at this concentration. For groups receiving PD-L1 treatment, anti-PD-L1 antibody (Bio X Cell, 10 mg/kg) was administered via intraperitoneal injection starting one day after PDT therapy and repeated daily for three consecutive days, for a total of three doses [37]. Tumor length and width were measured every other day for 14 days. At the end of the experiment, all mice were euthanized following humane endpoints guidelines. Major organs and tumor tissues were collected for subsequent analysis. The tissue samples were subjected to H&E staining to evaluate morphological changes, Ki67 staining to assess proliferation, and TUNEL staining to determine apoptosis status.

### 2.27 Tumor immune microenvironment analysis

Flow cytometry was performed to analyze the immune TME in C3H/He mice, focusing on macrophages, DCs, and effector CD8^+^ T cells. Four days post-treatment, tumors and spleens were harvested, dissociated in PBS, and tumor fragments were enzymatically digested using DNase, Liberase™ TL, and collagenase IV (5 mg/mL) at 37 °C for 1.5 h with agitation at 500 rpm. The cell suspensions were filtered through a 70 μm sieve, centrifuged, and resuspended in PBS with 5 % FBS. Spleen cells were obtained by grinding against a 70 μm sieve, followed by centrifugation and RBC lysis, after which cells were resuspended in PBS with 1 % FBS.

For macrophage M1 polarization analysis, cells were stained with 7-AAD viability dye and antibodies against CD45, CD11b, F4/80, and CD86. DC maturation was assessed using 7-AAD, CD45, CD11c, CD86, and CD80 antibodies. Effector CD8^+^ T cells were stained with 7-AAD, CD45, CD3, and CD8 antibodies. All staining steps were conducted at 4 °C in the dark.

For immunofluorescence analysis, 4 μm thick tumor sections were prepared. After deparaffinization and antigen retrieval using EDTA, sections were blocked and then treated with primary antibodies targeting CD86 and CD8. After washing, sections were treated with fluorescently labeled secondary antibodies, counterstained with DAPI, and mounted for FL microscopy.

Additionally, serum levels of TNF-α, IL-6, and IL-12 were analyzed using ELISA. Blood was collected via retro-orbital bleeding, allowed to clot, and centrifuged to obtain serum. Cytokine levels were quantified following the protocol, and absorbance was measured at 450 nm to calculate concentrations based on standard curves.

### 2.28 Abscopal therapy effect *in vivo*

To establish a contralateral tumor model, C3H/He mice were subcutaneously injected with 1 × 10^6^ SCC7 cells in 100 μL of saline in the right flank. Once tumors developed to 100 mm^3^, the mice were divided into different treatment groups. Treatments were administered accordingly, and four days post-treatment, 1 × 10^6^ SCC7 cells were subcutaneously injected into the left flank of each mouse. The size of the tumors on the left flank was measured every other day using calipers, and the measurements were recorded to monitor tumor growth kinetics. Fourteen days after the development of the tumors on the left flank, the tumors were harvested for size measurement. Additionally, 10 days after the formation of the contralateral tumor, abscopal tumor samples were processed for flow cytometry to assess macrophage polarization, DC maturation, and CD8^+^ T cell status.

### 2.29 The efficiency against tumor lung metastasis

C3H/He mice were subcutaneously injected with 1 × 10^6^ SCC7-LUC cells in 100 μL of saline into the right flank. Once the tumors developed to approximately 100 mm^3^, the mice were divided into different treatment groups and subjected to the respective treatments. Four days post-treatment, each mouse received an intravenous injection of 2 × 10^5^ SCC7-LUC cells via the tail vein. Two weeks later, the mice were injected intraperitoneally with luciferase substrate, and *in vivo* imaging was performed using a bioluminescence imaging system to assess lung metastasis. The lung tissues were then processed and stained with H&E to confirm and analyze the presence of metastatic lesions.

### 2.30 Survival analysis

C3H/He mice were subcutaneously injected with 1 × 10^6^ tumor cells in the right flank to establish tumors. Once tumors reached approximately 100 mm^3^, mice were divided into treatment groups. After receiving the respective treatments, mice were monitored daily for survival. Euthanasia was performed when tumors exceeded 2000 mm^3^, severe weight loss occurred, or significant distress was observed. Survival time was recorded, and Kaplan-Meier curves were generated to compare survival rates between groups.

### 2.31 Transcriptomic analysis

For transcriptomic analysis, samples were stored at −80 °C until processing. Total RNA was extracted and subjected to RNA sequencing to obtain the reference transcriptome. Differential gene expression analysis was performed to identify significantly differentially expressed genes between treatment groups. Functional enrichment analyses, including GO and KEGG pathway analyses, were conducted to interpret the biological significance of the differentially expressed genes. Additionally, immune infiltration analysis was performed to assess the immune cell composition within the TME. GSVA was employed to assess variations in immune-related gene sets across different groups, offering insights into the immune TME and potential mechanisms underlying the treatment responses.

### 2.32 Biocompatibility analysis

To evaluate the vitro biocompatibility of different nanomaterials, three cell lines (3T3, RAW264.7, SCC7) were treated with varying concentrations (up to 1000 μg/mL) of the nanomaterials, and cell viability was assessed using the CCK-8 assay. For the hemolysis assay, blood cells collected from C3H/He mice were incubated with nanomaterials at different concentrations for 30 min. The samples were then centrifuged to examine hemolysis.

To assess *in vivo* biocompatibility, blood samples were obtained for biochemical analysis and routine blood tests, while major organs were excised for histological analysis. These tests were conducted to assess cytotoxicity and overall biocompatibility of the protein vesicle.

### 2.33 Statistical analysis

Statistical analysis was conducted using GraphPad Prism and R software. To evaluate statistical significance, Student's t-test and one-way ANOVA were applied. Tukey's post-hoc test was used for ANOVA comparisons. A p-value threshold of less than 0.05 was used to define statistical significance, with specific levels denoted as ∗p < 0.05, ∗∗p < 0.01, ∗∗∗p < 0.001, and ∗∗∗∗p < 0.0001. Non-significant findings were labeled as "ns".

## 3 Results and discussion

### 3.1 Synthesis and characterization of the protein vesicle

The fabrication of the carrier-free flexible dual-enzyme protein vesicle involved a stepwise synthesis process. Initially, MSNs were synthesized and surface-functionalized by adsorption of PEI followed by crosslinking with GA to stabilize the coating. Subsequently, HSA and two natural enzymes, CAT and LOX, were covalently conjugated onto the GA-modified MSNs at varying ratios (4:1, 3:1, 2:1, 1:1, 1:2) via amide bond formation. The silica scaffold was then etched away using HF, yielding the carrier-free flexible protein vesicle (HSA/CAT/LOX). Finally, the photosensitizer IR808 was conjugated to the protein vesicle, completing the formation of HSA/CAT/LOX@IR808.

**Fig. 1:**
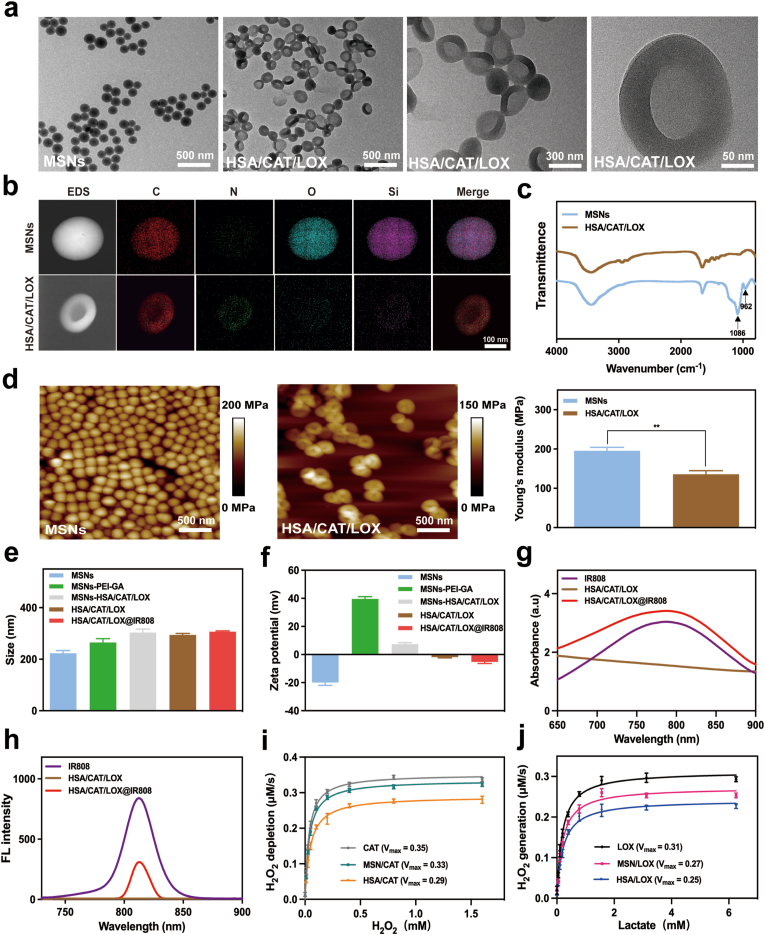
Characterization of the carrier-free dual-enzyme protein vesicle. (a) TEM images, and (b) element mapping images of MSNs and the etched HSA/CAT/LOX. (c) FT-IR spectra analyses of post-etching bond changes. (d) AFM analysis and Young's modulus changes of MSNs and the etched HSA/CAT/LOX. (e, f) DLS analyses of particle size and Zeta potential. (g) UV–vis spectrum analyses of IR808 incorporation. (h) FL spectra demonstrating IR808 conjugation. (i, j) Michaelis-Menten curves of CAT and LOX before and after etching. Data are presented as mean ± SD. Statistical analyses were performed using Student's t-test.

TEM analysis showed that the morphology of the synthesized MSNs was uniform and spherical (Fig. 1a). After encapsulating HSA, CAT, and LOX, and etching with HF, TEM images demonstrated that all the etched protein vesicles, synthesized with different CAT-LOX ratios, developed a hollow interior and a wrinkled surface (Fig. 1a and Fig. S1). Furthermore, after IR808 conjugation, the HSA/CAT/LOX@IR808 maintained their hollow vesicle morphology, suggesting good structural stability (Fig. S2). Element mapping further confirmed successful etching, showing a consistent presence of C and N, while the disappearance of O and Si indicated the removal of the silica-based scaffold (Fig. 1b). Quantitative 10.13039/100004679EDS analysis further supported this observation, with a marked decrease in Si and O content after HF etching, where O content decreased from 14.6 % to 0.5 %, and Si content decreased from 30.6 % to 0.3 % (Fig. S3). FT-IR analysis also supported this, as evidenced by the disappearance of the Si-O-Si stretching peak (1086 cm^−1^) and the Si-H bending peak (962 cm^−1^), confirming the successful etching process (Fig. 1c). Young's modulus results indicated that the MSNs before etching has a Young's modulus of 196 ± 9 MPa, while the etched HSA/CAT/LOX has a Young's modulus of 136 ± 10 MPa, suggesting that the etching process significantly reduces the rigidity, enhancing the flexibility (Fig. 1d).

Dynamic light scattering (DLS) measurements of HSA/CAT/LOX@IR808 samples indicated that they were 310 ± 20 nm in size, with good dispersion, and a Zeta potential of −4 mV (Fig. 1e and f). The UV–vis spectrum displayed a strong absorption peak at 800 nm, confirming the efficient loading of IR808 (Fig. 1g). Additionally, FL spectra indicated that the protein vesicle exhibited excellent NIR FL properties, supporting their potential for use in NIR-based applications (Fig. 1h). The loading efficiency of each component in the HSA/CAT/LOX@IR808 was determined as follows: 8.1 mg of HSA (EE = 80.7 %, LC = 51.0 %), 2.4 mg of CAT (EE = 78.9 %, LC = 15.0 %), 1.3 mg of LOX (EE = 66.7 %, LC = 8.4 %), and 1.3 mg of IR808 (EE = 65.0 %, LC = 8.2 %) (Supplementary Table S1). The final loading ratio of HSA:CAT:LOX:IR808 was 8.1:2.4:1.3:1.3. HSA/CAT/LOX@IR808 remained well-dispersed in PBS (pH 7.4) for 7 days, indicating good solubility and stability (Fig. S4). TEM images demonstrated that the protein vesicle retained the morphology with stable DLS and Zeta potential even after 30 days of storage (Fig. S5).

### 3.2 Enzyme activity assessment

Building upon these characterization results, we next evaluated the enzymatic activity of the system, specifically focusing on the effect of HF etching on the functionality of CAT and LOX.

To assess the activity of CAT, samples were placed in a water bath at 37 °C for 30 min of incubation with or without H_2_O_2_. Optical images were taken to observe bubble formation, indicating oxygen release. Significant bubble formation was observed in the HSA/CAT/LOX@IR808 group upon H_2_O_2_ addition, confirming the activity of CAT (Fig. S6).

The enzyme activity of CAT and LOX before and after HF etching was evaluated by measuring the V_max values for pure CAT, MSN/CAT (pre-etching), and HSA/CAT (post-etching); and for pure LOX, MSN/LOX (pre-etching), and HSA/LOX (post-etching). The V_max values for CAT were 0.35 (pure CAT), 0.33 (MSN/CAT), and 0.29 (HSA/CAT), while for LOX they were 0.31 (pure LOX), 0.27 (MSN/LOX), and 0.25 (HSA/LOX). These results indicate that the etching process caused a slight reduction in enzyme activity, but more than 80 % of the activity was preserved, confirming that the optimized 5 % HF etching process maintains enzyme functionality. The detailed Michaelis-Menten curves are shown in Fig. 1i and j.

### 3.3 Assessment of synergistic enzyme cascade amplification activity

Building upon the enzymatic activity data, we next evaluated the synergistic enzyme cascade amplification effect and compared enzyme activity across different enzyme ratios. To achieve this, we monitored changes in lactate, pH, and H_2_O_2_ levels following lactate addition in the various experimental groups.

**Fig. 2:**
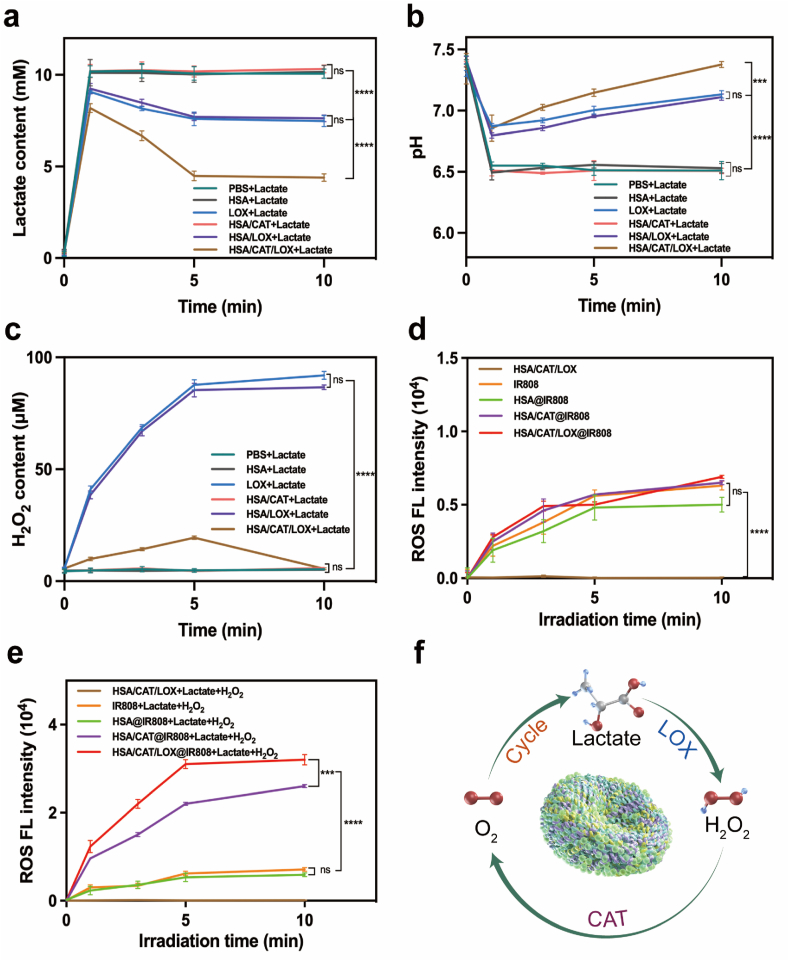
Characterization of the enzyme-linked amplification mechanism in the protein vesicle. (a, b, c) Changes in lactate, pH, and H_2_O_2_ levels after the addition of lactate, illustrating the effects of the dual-enzyme cascade. (d) ROS production over time under light irradiation. (e) ROS production after adding lactate and H_2_O_2_. (f) Schematic illustration of the dual-enzyme cascade mechanism. Data are presented as mean ± SD. Statistical analyses were performed using one-way ANOVA with Tukey's post hoc tests.

In the PBS + Lactate group, lactate levels increased significantly after lactate addition, as expected. In the free LOX group, lactate concentrations decreased rapidly due to LOX-mediated oxidation, serving as a benchmark for LOX activity. In contrast, the etched HSA/LOX group exhibited a similar decrease in lactate concentration, confirming that LOX retained high catalytic activity. This decrease in lactate levels was accompanied by a moderate pH increase, reaching a pH of approximately 7.0, but failing to return to the original pH of 7.4. In the dual-enzyme group (HSA/CAT/LOX), the reduction in lactate concentration was significantly greater compared to the single-enzyme groups. This phenomenon can be attributed to the cascade effect: LOX catalyzes the conversion of lactate, generating H_2_O_2_ as a byproduct, which then serves as a substrate for CAT to produce oxygen. The generated oxygen further facilitates the LOX-catalyzed lactate consumption, creating a positive feedback loop that amplifies the overall enzymatic reaction. As a result, pH levels increased more significantly in this group, eventually returning to near the original pH of 7.4 (Fig. 2a and b). With respect to H_2_O_2_ levels, the free LOX group and the etched HSA/LOX group showed a steady increase in H_2_O_2_ production over time, reaching a plateau of approximately 80 μM due to the continuous generation of H_2_O_2_ by LOX. However, in the HSA/CAT/LOX group, H_2_O_2_ levels only exhibited slight increases at the 1, 3, and 5-min marks, followed by a decline as CAT consumed H_2_O_2_. By the 10-min mark, H_2_O_2_ had been almost completely depleted, approaching the levels observed in the PBS control group (Fig. 2c). The five different ratios (4:1, 3:1, 2:1, 1:1, 1:2) were tested, and the 3:1 CAT-LOX ratio was found to exhibit the most significant changes in lactate consumption, pH increase, and H_2_O_2_ depletion (Fig. S7a–c). This suggests that the 3:1 ratio optimizes the synergistic interaction between CAT and LOX, leading to superior catalytic performance.

Overall, these findings confirmed the retained biological activity of the synergistic behavior of this protein vesicle system, demonstrating its potential ability to enhance lactate consumption, reduce acidity, and maintain balanced H_2_O_2_ levels.

### 3.4 Enzyme-linked amplification enhances ROS generation

After successfully having confirmed the enzyme-cascade amplification of the prepared protein vesicle, we investigated whether this interaction was capable of enhancing ROS generation (^1^O_2_) under NIR irradiation. ^1^O_2_, the primary and most cytotoxic ROS species in photodynamic reaction, was quantified using the specific fluorescent probe SOSG [[9], [10], [11], [12]]. Fig. 2d illustrates a time-dependent rise in ROS production across all groups containing the photosensitizer IR808 upon irradiation with light at 808 nm. When lactate and H_2_O_2_ were added to simulate tumor extracellular conditions, ROS production significantly increased, particularly in the HSA/CAT/LOX@IR808 group. The presence of CAT improved the hypoxic environment by breaking down H_2_O_2_ into oxygen, enhancing ROS generation. The addition of LOX further amplified this effect through a cascade reaction, resulting in the highest ROS levels (Fig. 2e). These results demonstrated that the vesicle system significantly enhances ROS generation through the enzymatic cascade amplification mechanism (Fig. 2f).

To determine the optimal CAT-LOX ratio for maximum ROS generation, we tested protein vesicles synthesized with varying enzyme ratios. Among these ratios, the 3:1 CAT-LOX ratio produced the highest ROS levels, demonstrating the most efficient enzymatic cascade and oxidative stress enhancement (Fig. S7d). Therefore, the 3:1 CAT-LOX ratio was selected for subsequent experiments.

### 3.5 Targeting capability and biodistribution analyses

We next focused on evaluating its targeting capability, which is critical for subsequent cellular and animal studies. For these experiments, the SCC7 HNSCC cell line was used. In animal studies, SCC7 cells were implanted in nude mice to assess *in vivo* targeting efficiency. Tumor FL was quantified in Aniview by manually drawing ROIs. The software calculated average intensity, which was used for group comparison (Fig. S8).

**Fig. 3:**
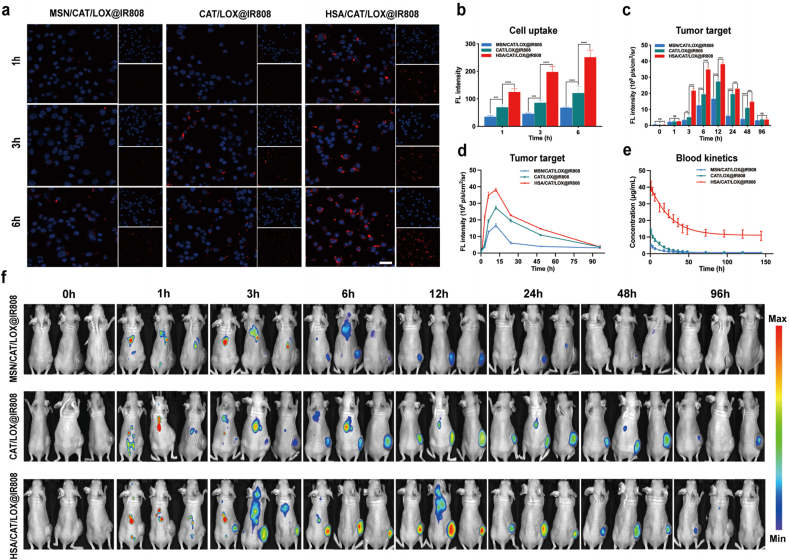
Targeting efficiency and biodistribution of MSN/CAT/LOX@IR808, CAT/LOX@IR808, and HSA/CAT/LOX@IR808. (a, b) SCC7 cellular internalization analysis of different nanomaterials at different time points. Scale bar: 50 μm. (c, d, f) *In vivo* FL imaging of SCC7 tumor-bearing nude mice, showing FL signal distribution over time in different treatment groups (n = 3). (e) Pharmacokinetic analysis shows that the flexible HSA-based vesicle has much longer blood circulation than the rigid counterparts. Data are presented as mean ± SD. Statistical analyses were performed using one-way ANOVA with Tukey's post hoc tests.

Biological electron microscopy (Fig. S9) and FL microscopy experiments for cellular internalization (Fig. 3a and b) demonstrated that CAT/LOX@IR808 exhibited higher cellular uptake in SCC7 HNSCC cell line compared to MSN/CAT/LOX@IR808, highlighting the critical role of flexibility in enhancing cellular delivery [[32], [33], [34]]. Building upon this, HSA/CAT/LOX@IR808 exhibited the highest internalization efficiency, significantly surpassing CAT/LOX@IR808. HSA contributes to enhanced tumor targeting through multiple mechanisms, including gp60-mediated transcytosis and SPARC (secreted protein, acidic and rich in cysteine)-mediated retention. SPARC is frequently overexpressed in the tumor stroma and has a high binding affinity for albumin, thereby facilitating the accumulation of albumin-based nanocarriers within the tumor interstitium. This interaction enhances both the retention and localization of HSA-containing nanoparticles in tumor tissues [[34], [35], [36]]. The combined effects of flexibility and HSA incorporation synergistically enhance the cellular uptake of HSA/CAT/LOX@IR808.

*In vivo* targeting efficiency was assessed in nude mice implanted with SCC7 cells via FL imaging (Fig. 3c,d,f). Compared to MSN/CAT/LOX@IR808, CAT/LOX@IR808 exhibited enhanced tumor accumulation, benefiting from its flexible structure. Notably, HSA/CAT/LOX@IR808 achieved the highest tumor accumulation, with peak FL intensity at 12 h post-injection. This superior targeting efficiency highlights the combined impact of flexibility, which improves tumor penetration, and HSA, which facilitates both gp60-mediated transendothelial transport and SPARC-enhanced tumor retention.

Pharmacokinetic analysis revealed that the area under the curve (AUC_0-∞_) values for MSN/CAT/LOX@IR808, CAT/LOX@IR808, and HSA/CAT/LOX@IR808 were 0.14, 0.25, and 2.3 mg/mL/h respectively (Fig. 3e). These results demonstrate that the flexible, HSA-based vesicle exhibits significantly prolonged blood circulation compared to the more rigid counterparts. This enhancement is attributed to the combined effects of the flexibility, which improves deformability, and HSA incorporation, which promotes systemic retention through albumin-mediated transport and tumor-targeting pathways [[34], [35], [36]]. FL signals in major organs were assessed for HSA/CAT/LOX@IR808, peaking within 1–3 h post-injection. The signals gradually decreased and were nearly eliminated within 4 days, indicating efficient clearance and minimizing potential toxicity (Fig. S10). Although no obvious short-term toxicity was detected, potential chronic toxicity, immunogenicity, and off-target effects warrant further study. Future work will aim to improve pharmacokinetics and tumor specificity while reducing systemic exposure.

### 3.6 Dual-targeted modulation of hypoxia and lactate

**Fig. 4:**
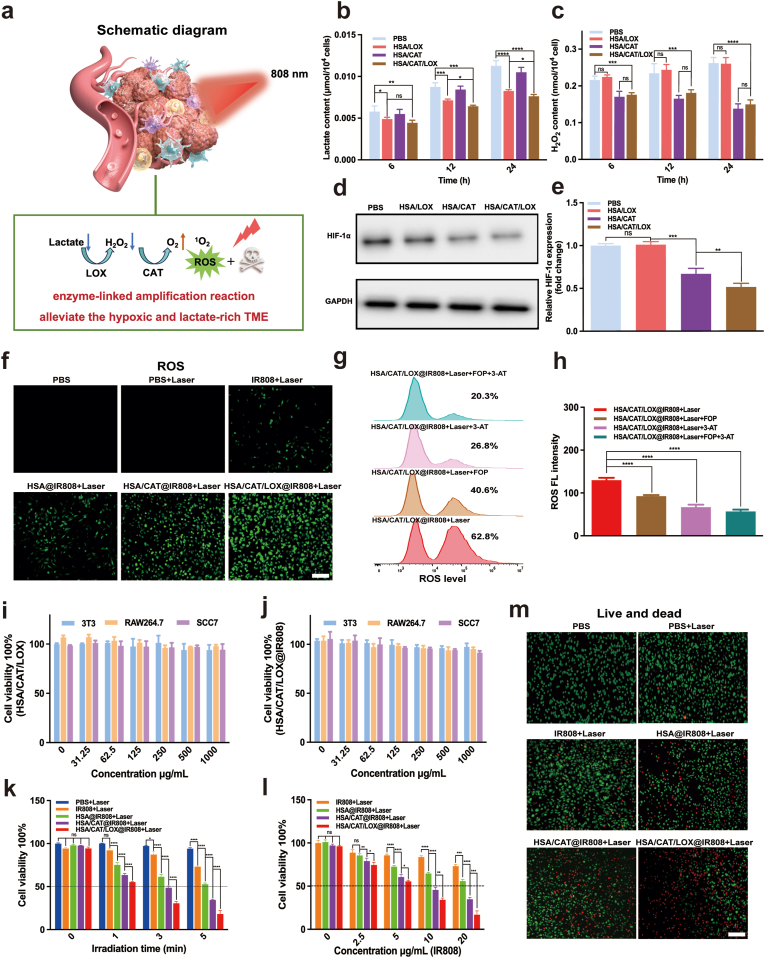
The synergistic protein vesicle alleviates intracellular hypoxia and lactate accumulation, thereby enhancing ROS generation and promoting cell killing. (a) Schematic diagram. (b, c) Analysis of intracellular lactate accumulation and H_2_O_2_ levels in SCC7 cells after treatment different treatment. (d, e) Assessment of intracellular HIF-1α expression within different groups. (f) ROS generation upon light exposure. Flow cytometry (g) and FL quantitative analysis (h) of ROS signal levels after treatment with the LOX inhibitor FOP, the CAT inhibitor 3-AT, or FOP + 3-AT. (i, j) Biocompatibility analyses of HSA/CAT/LOX and HSA/CAT/LOX@IR808 at various concentrations. CCK8 assay (k, l) and cell viability/death staining (m) demonstrating cytotoxicity in different groups subjected to 808 nm irradiation. Scale bar: 50 μm. Data are presented as mean ± SD. Statistical analyses were performed using one-way ANOVA with Tukey's post hoc tests.

Next, we evaluated the enzyme cascade effect of the protein vesicle on regulating hypoxia and lactate accumulation in SCC7 tumor cells (Fig. 4a). Intracellular lactate and H_2_O_2_ levels were assessed at 6, 12, and 24 h post-treatment with different treatments. In the HSA/LOX group, intracellular lactate levels showed a significant decrease compared to the control group, indicating efficient LOX-mediated lactate catalysis (Fig. 4b). However, this process was accompanied by the accumulation of H_2_O_2_, a byproduct of LOX-catalyzed lactate oxidation (Fig. 4c). In contrast, the dual-enzyme group (HSA/CAT/LOX) not only exhibited a more pronounced reduction in lactate levels but also effectively scavenged the accumulated H_2_O_2_ due to the introduction of CAT, highlighting the enhanced catalytic efficiency provided by the dual-enzyme system (Fig. 4b and c).

To further understand the impact on the hypoxic microenvironment, HIF-1α levels in SCC7 cells were measured via WB (Fig. 4d and Fig. S11) and qPCR (Fig. 4e). HIF-1α is crucial as it is a primary regulator of hypoxia. These analyses demonstrated that HSA/CAT alleviated intracellular hypoxia, resulting in a marked reduction in HIF-1α expression. The inclusion of LOX (HSA/CAT/LOX) in the protein vesicle further decreased HIF-1α expression. This enhanced effect was attributed to the enzyme-linked amplification mechanism, leading to more effective hypoxia relief.

### 3.7 The protein vesicle enhances ROS generation through cascade reactions

Building on the successful alleviation of hypoxia and lactate, we further investigated the ability of the prepared protein vesicle to enhance ROS production through enzyme cascade reactions.

To demonstrate the role of enzyme-linked amplification in the enhancement of ROS production, six different groups were assessed in SCC7 cells: PBS, PBS + Laser, IR808+Laser, HSA@IR808+Laser, HSA/CAT@IR808+Laser, and HSA/CAT/LOX@IR808+Laser. Both FL imaging (Fig. 4f and Fig. S12a) and flow cytometry data (Fig. S12b and c) showed minimal ROS production in the PBS and PBS + Laser groups, whereas a slight increase was apparent in the IR808+Laser group. ROS levels progressively increased with the addition of enzyme components, with peak levels in the HSA/CAT/LOX@IR808+Laser group, highlighting the role of enzyme-linked amplification in ROS generation enhancement. Additionally, similar trends were observed in HN6 and FaDu cells (Fig. S13), further supporting the enzyme amplification effect across different HNSCC cell lines.

Further validation of this enzyme-linked ROS amplification was achieved by introducing two enzyme inhibitors: α-Fluoro-β-oxopropionate (FOP, LOX inhibitor), 3-Amino-1,2,4-triazole (3-AT, CAT inhibitor), and their combination. FL imaging and flow cytometry data (Fig. 4g,h and Fig. S14) demonstrated that combined FOP + 3-AT treatment led to the most pronounced decrease in ROS production, surpassing the effects of either inhibitor individually. These findings underscore that the enzyme cascade plays the pivotal role in amplifying ROS generation.

### 3.8 Effects of the protein vesicle on cell proliferation, migration, and invasion

Subsequently, we evaluated the antitumor efficacy of IR808-loaded nanovesicles with laser irradiation on SCC7 cells, including their effects on cell proliferation, migration, and invasion capabilities. First, the biocompatibility of the protein vesicle, including HSA/CAT/LOX and HSA/CAT/LOX@IR808, was evaluated. At concentrations up to 1000 μg/mL, neither formulation had any significant toxicity when used to treat SCC7, RAW264.7, or 3T3 cells (Fig. 4i and j). The CCK-8 assay demonstrated a time-dependent and IR808 concentration-dependent increase in cytotoxicity across all experimental groups (Fig. 4k and l). While the IR808+Laser group showed moderate cell damage, the HSA@IR808+Laser group exhibited significantly enhanced cytotoxicity due to improved targeting efficiency. Notably, the HSA/CAT@IR808+Laser and HSA/CAT/LOX@IR808+Laser groups displayed the most pronounced cell-killing effects, which can be attributed to the enzyme cascade amplification facilitating more efficient ROS generation. These findings were consistently supported by cell viability/death staining (Fig. 4m and Fig. S15) and flow cytometry analysis (Fig. S16), with the HSA/CAT/LOX@IR808+Laser group showing the highest apoptosis rate, likely resulting from the synergistic effects of LOX and CAT in enhancing ROS production and triggering intrinsic apoptosis pathways. To further confirm the therapeutic efficacy across different tumor types, CCK-8 assays were also performed in human HNSCC cell lines HN6 and FaDu (Fig. S17). The vesicles exhibited effective cytotoxicity across all tested cell lines, with IC_50_ values of 5.748 μg/mL for SCC7, 3.085 μg/mL for HN6, and 2.891 μg/mL for FaDu, respectively (Fig. S18), supporting the potential translational applicability of this strategy.

Colony formation assays (Fig. S19) revealed a significant reduction in colony numbers in the IR808+Laser group compared to the PBS + Laser group, with enzyme-loaded nanomaterials further suppressing proliferation. Wound healing assays (Fig. S20) demonstrated that the enzyme-loaded vesicle, particularly HSA/CAT/LOX@IR808+Laser, markedly inhibited SCC7 cell migration, showing minimal wound closure. Similarly, transwell migration (Fig. S21) and invasion assays (Fig. S22) confirmed that the enzyme-loaded vesicle, especially HSA/CAT/LOX@IR808+Laser, significantly hindered migration and invasion. These results highlight the protein vesicle's effectiveness in inhibiting SCC7 cell proliferation, migration, and invasion.

### 3.9 ICD induction and enhanced macrophage phagocytosis

**Fig. 5:**
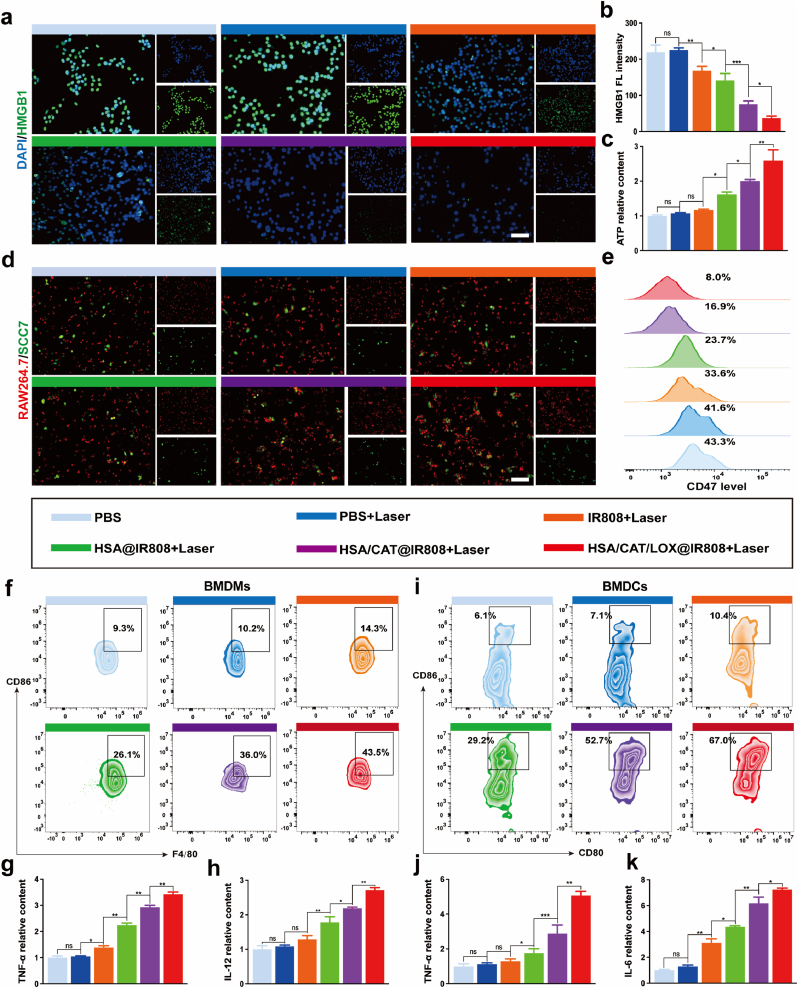
Overview of the effects of treatment with the protein vesicle on immune responses. (a, b) FL analysis of nuclear HMGB1 levels. (c) ELISA analysis of extracellular ATP levels. (d) FL imaging showing macrophage phagocytosis under conditions of different treatment (red: Raw 264.7 cells, green: SCC7 cells). (e) Analysis of CD47 expression using flow cytometry following different treatment. (f) Flow cytometry analysis of M1 macrophage polarization. (g, h) ELISA analyses of cytokine quantities (TNF-α, IL-12) in supernatants from BMDMs post-treatment. (i) Examining DC maturation through flow cytometry. (j, k) ELISA analyses of BMDCs cytokine quantities (TNF-α, IL-6) in supernatants from BMDCs post-treatment. Scale bar: 50 μm. Data are presented as mean ± SD. Statistical analyses were performed using one-way ANOVA with Tukey's post hoc tests. (For interpretation of the references to colour in this figure legend, the reader is referred to the Web version of this article.)

We investigated ICD induction by the protein vesicle through ROS amplification. Reduced nuclear HMGB1 levels (ICD biomarker) confirmed NIR-induced ICD [[38], [39], [40]], with HSA/CAT/LOX@IR808+Laser showing maximal HMGB1 depletion due to oxygen-ROS synergy (Fig. 5a and b). Concurrently, extracellular ATP significantly evaluated (Fig. 5c), establishing chemotactic "find-me" signals for immune cell recruitment [41].

After ICD induction, we explored its impact on macrophage phagocytosis, focusing on CD47, the cellular "don't eat me" signal [[41], [42], [43], [44], [45], [46]]. Flow cytometry showed a significant decrease in CD47-positive cells after treatment (Fig. 5e and Fig. S23). CD47 expression dropped from 43.3 % in the PBS group to 8.0 % in the HSA/CAT/LOX@IR808+Laser group. FL co-localization analysis further demonstrated that enhanced macrophage phagocytosis across treatment groups, with the HSA/CAT/LOX@IR808+Laser group achieving the highest phagocytosis index of 70 % (Fig. 5d and Fig. S24). It is possible that ROS generated during PDT may trigger cellular stress responses, leading to the downregulation of CD47 expression, a mechanism that has been observed in previous studies under oxidative stress conditions [[43], [44], [45]]. This reduction in CD47 expression could contribute to the enhanced macrophage-mediated phagocytosis.

### 3.10 Enhanced PDT induces M1 macrophage polarization and DC maturation

To further assess the impact of enhanced PDT treatment on immune cells, supernatants from treated tumor cells were co-incubated with BMDMs and BMDCs for 48 h. Flow cytometry and ELISAs were used to analyze immune cell status and cytokine levels after such treatment (Fig. S25).

Flow cytometry results (Fig. 5f and Fig. S26a) revealed that the IR808+Laser group exhibited a slight increase in the M1 polarization of BMDMs. Notably, the HSA@IR808+Laser group exhibited a significant rise in M1 polarization, which was further significantly amplified in the HSA/CAT@IR808+Laser and HSA/CAT/LOX@IR808+Laser groups. These findings suggest that the enzyme-loaded protein vesicle enhance the efficacy of PDT, readily activating the immune response. Consistently, ELISAs (Fig. 5g and h) revealed elevated levels of TNF-α and IL-12 production following treatment, particularly in the enzyme-loaded groups, further supporting the observed immune activation.

Similarly, flow cytometry results (Fig. 5i and Fig. S26b) showed a marked increase in BMDC maturation in the HSA@IR808+Laser group compared to the PBS and PBS + Laser groups. This effect was even more pronounced in the HSA/CAT@IR808+Laser and HSA/CAT/LOX@IR808+Laser groups. ELISAs (Fig. 5j and k) also confirmed significant increases in TNF-α and IL-6 production, highlighting the induction of a robust immune response in these groups.

Flow cytometry data revealed the significant upregulation of PD-L1 across all the treatment groups compared to PBS controls, with the highest increase being observed in the HSA/CAT/LOX@IR808+Laser group (Fig. S27). This observation highlights the potential benefits of combining PD-L1 inhibitors with this enhanced PDT therapy to counteract immune suppression and synergistically enhance therapeutic outcomes [47,48].

### 3.11 *In vivo* evaluation of the antitumor efficacy

**Fig. 6:**
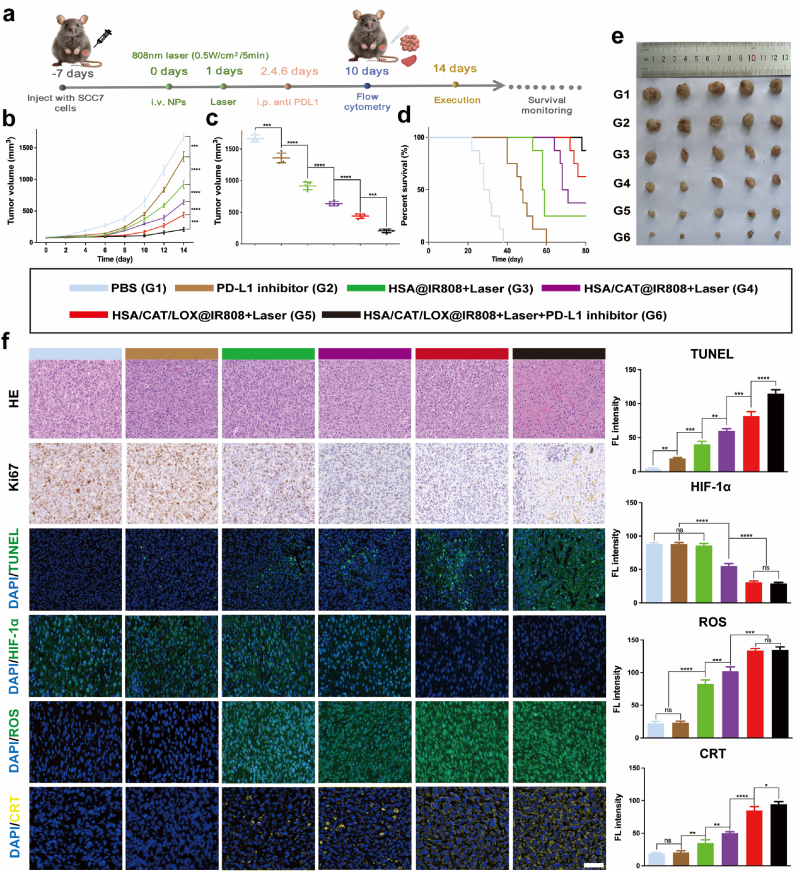
Anti-tumor enhancement of the protein vesicle with immunotherapy. (a) Schematic diagram of the therapeutic schedule. (b–e) Tumor growth curves, final tumor volumes, and survival analysis in SCC7 tumor-bearing mice following various treatments. (f) H&E, Ki67, TUNEL, HIF-1α, ROS, and CRT staining and FL intensities quantification of tumor tissues in different groups. Scale bar: 50 μm. Data are presented as mean ± SD. Statistical analyses were performed using one-way ANOVA followed by Tukey's post hoc tests.

We next evaluated the overall *in vivo* antitumor efficacy of nanovesicle-mediated PDT, and its combination with immune checkpoint blockade (ICB) (Fig. 6a). Mice were divided into six groups: PBS (G1), PD-L1 inhibitor (G2), HSA@IR808+Laser (G3), HSA/CAT@IR808+Laser (G4), HSA/CAT/LOX@IR808+Laser (G5), and HSA/CAT/LOX@IR808+Laser + PD-L1 inhibitor (G6). Tumor growth was inhibited to varying degrees in all treatment groups compared to G1, with tumor size being monitored over 14 days (Fig. 6b,c and Fig. S28) and measured after dissection (Fig. 6e). The G2 group showed significant tumor growth reduction, although tumor volumes still exceeded 1000 mm^3^ after 14 days. In contrast, IR808-loaded protein vesicle (G3-G5) significantly slowed tumor growth, with inhibition progressively increasing due to the enhanced PDT efficacy. The combination treatment (G6) yielded the most substantial tumor inhibition, suggesting a synergistic interaction between PDT and PD-L1 inhibition. Body weight remained stable across all groups (Fig. S29). Histological analysis (Fig. 6f) confirmed that G6 had the lowest tumor cell proliferation (Ki67 staining) and the highest levels of apoptosis (TUNEL staining). However, the *in vivo* evaluation was limited to a single murine HNSCC model, which may affect the generalizability of the findings. Additional validation in other animal models is needed to better assess translational potential.

Survival analyses (Fig. 6d) showed that animals in G1 exhibited the shortest median survival time (35 days), with moderate improvements in G2 (55 days). Nanoparticle-treated groups (G3-G5) demonstrated progressively better survival outcomes, with G5 showing a substantial increase. The combination group (G6) exhibited the most significant improvement, with 87.5 % of C3H/He mice surviving over 80 days.

### 3.12 Lactate consumption and hypoxia alleviation *in vivo*

To assess *in vivo* metabolic changes, tumor tissues were collected after treatment and analyzed for lactate and H_2_O_2_ content. As shown in Fig. S30a, the HSA/LOX group showed a significant decrease in intratumoral lactate levels, indicating effective LOX-mediated lactate oxidation. However, this was accompanied by elevated H_2_O_2_ accumulation (Fig. S30b). In contrast, the HSA/CAT/LOX group exhibited the most pronounced reduction in lactate along with markedly lower H_2_O_2_ levels, confirming that the dual-enzyme system efficiently consumes lactate while decomposing excess H_2_O_2_ through CAT activity.

Photoacoustic imaging was employed to measure the real-time changes in tumor blood oxygen saturation. As shown in Fig. S31, tumor oxygen saturation in the G1-G3 groups was approximately 60 %, consistent with high levels of HIF-1α expression observed in these groups (Fig. 6f). In contrast, the G4 group exhibited a significant increase in blood oxygen saturation, which correlated with a reduction in HIF-1α expression. Groups G5 and G6 showed even higher oxygen saturation, and correspondingly, the expression of HIF-1α was further reduced, highlighting the enhanced hypoxia relief achieved through the synergistic enzyme cascade. This indicates that the dual-enzyme system can more effectively reduce HIF-1α levels and modulate the hypoxic TME *in vivo*.

### 3.13 ROS generation, and ICD induction *in vivo*

ROS generation in tumors was assessed. The G1 and G2 groups displayed minimal ROS production, with increased levels in the G3 group. In the G4, G5, and G6 groups, ROS production was further elevated, reflecting the enhanced catalytic activity of CAT. Among these, the G5 and G6 groups exhibited the most substantial ROS levels, indicating that the synergistic dual-enzyme cascade in these groups effectively amplified ROS production (Fig. 6f).

FL analyses also revealed increased CRT levels in the G3 group, indicating the induction of ICD during PDT. In the G4 and G5 groups, CRT expression further increased due to the synergistic enzyme cascade, which amplified ICD by enhancing ROS generation. The most pronounced CRT expression was observed in the G6 group, highlighting the synergistic effect of combining PDT with immunotherapy as a means of inducing ICD (Fig. 6f).

### 3.14 Remodeling the immunosuppressive TME in tumor-bearing mice

To further clarify the mechanisms responsible for the observed antitumor effects documented above, we examined how the enzyme-loaded protein vesicle influence the immunosuppressive TME by promoting immune activation [[49], [50], [51], [52]].

**Fig. 7:**
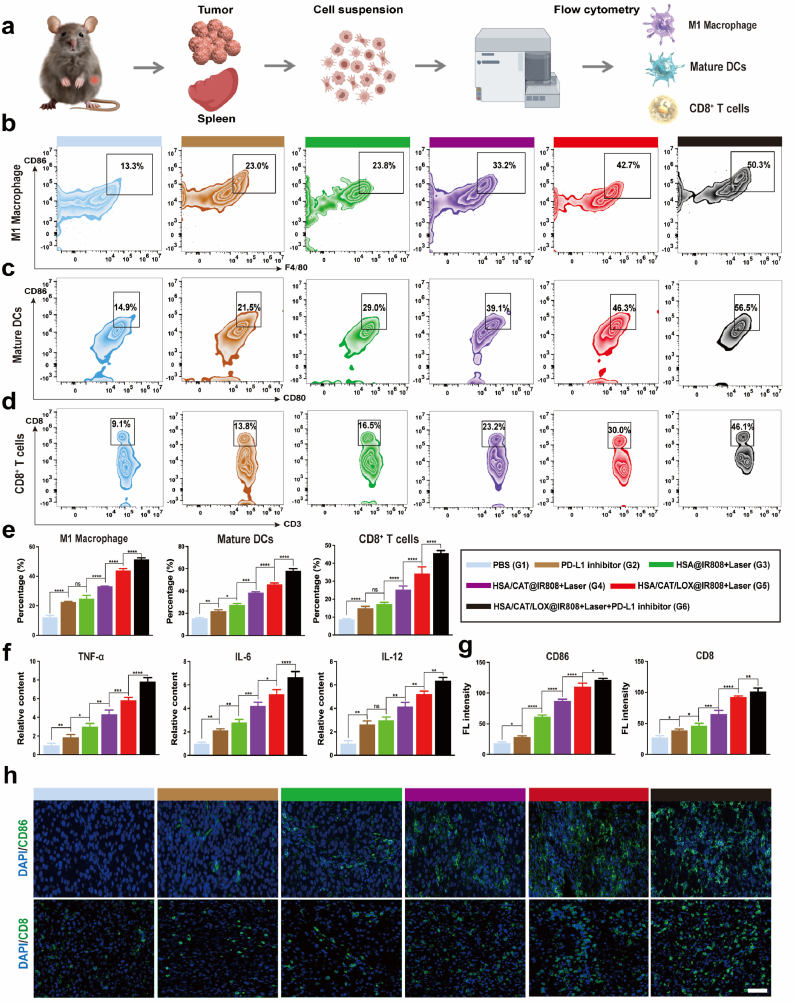
Immunomodulatory effects following different treatments. (a) Schematic of the experimental setup. (b, c, d) Flow cytometric analysis of M1 macrophages, DC maturation, and CD8^+^ T cells in tumor tissues. (e) Quantitative comparisons of M1 macrophages, DC maturation, and CD8^+^ T cells across treatment groups in tumor tissues. (f) Quantitative comparisons of serum cytokines (TNF-α, IL-6, IL-12) across treatment groups. (g, h) Immunofluorescence analysis of CD86 and CD8 expression in tumor tissues. Scale bar: 50 μm. Data are presented as mean ± SD. Statistical analyses were performed using one-way ANOVA with Tukey's post hoc tests.

Flow cytometry analyses of the spleen and tumor in C3H/He mice revealed significant changes in macrophage polarization, DC maturation, and CD8^+^ T cell infiltration across treatment groups (Fig. 7a). The G2 group, which underwent PD-L1 blockade treatment, exhibited notable increases in M1 polarization, DC maturation, and CD8^+^ T cell infiltration. This effect is likely due to the immune-activating properties of PD-L1 inhibition. The G3 group (HSA@IR808) also showed significant immune activation, including increases in both M1 polarization, DC maturation, and CD8^+^ T cell infiltration attributable to PDT-induced immunogenic effects. These effects were more noticeable in the G4 and G5 groups due to enzyme-mediated improvements in the hypoxic and lactate-rich TME, which amplified PDT efficacy and further activated the immune system. The combination group (G6) showed the most substantial effects, all of which were significantly higher than in the individual treatment groups (Fig. 7b–e and Fig. S32). ELISA results provided additional support for these findings as evidenced by increased amounts of pro-inflammatory cytokines like TNF-α, IL-6, and IL-12, in the G4, G5, and G6 groups, highlighting the enhanced immune activation (Fig. 7f). FL staining for CD86 and CD8 in tumor tissue samples supported these observations (Fig. 7g and h).

### 3.15 Contralateral tumor growth and lung metastasis inhibition

**Fig. 8:**
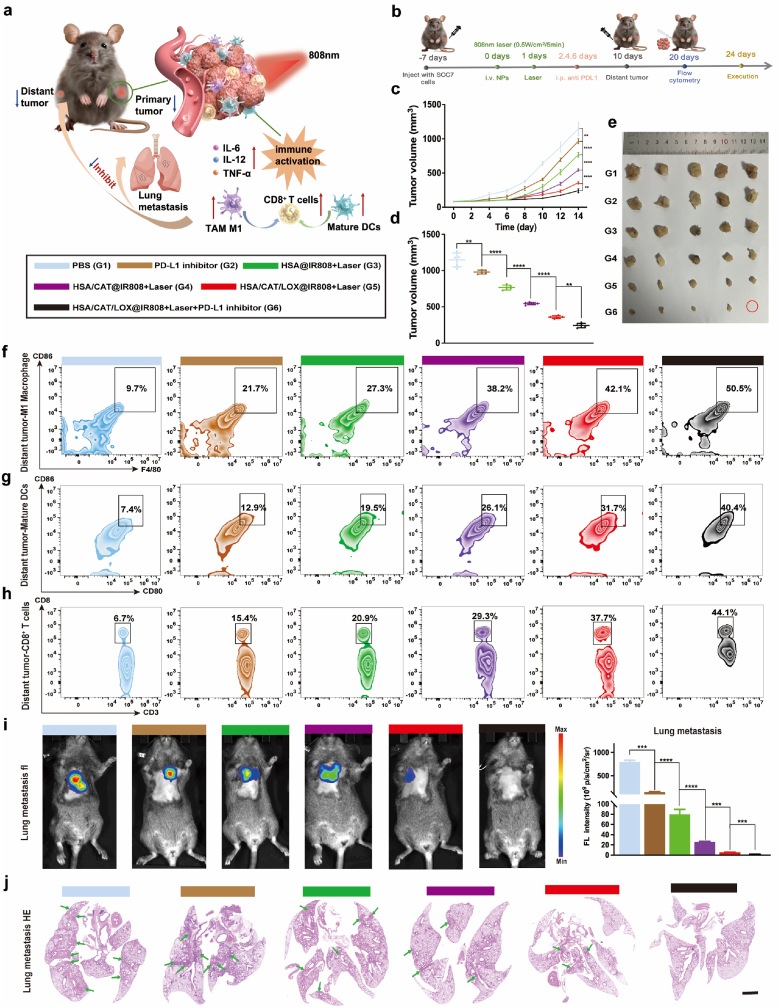
Enhanced systemic antitumor immunity suppresses contralateral tumors and prevents lung metastasis. (a) Flowchart illustrating the immune response post-treatment in C3H/He mice. (b) Schematic representation of the experimental protocol for contralateral tumor treatment. (c–e) Monitoring of contralateral tumor size and final tumor size at the end of the observation period. (f–h) Flow cytometric analysis of M1 macrophage polarization, DC maturation, and CD8^+^ T cell populations in contralateral tumors. (i, j) Bioluminescence imaging and lung H&E staining of lung metastases in C3H/He mice. Scale bar: 2 mm. Data are presented as mean ± SD, with statistical analysis performed using one-way ANOVA and Tukey's post hoc test.

Building on the immune remodeling observed within the primary TME, we next sought to determine whether this immune activation strategy could exert systemic effects by establishing contralateral tumor and lung metastasis models (Fig. 8a).

Four days post-treatment of the primary tumor, contralateral tumors were induced and monitored for 14 days (Fig. 8b). Tumor size progressively decreased from groups G4 to G6, with the G6 group showing the smallest tumors, indicating the most significant inhibition (Fig. 8c–e and Fig. S33). The combination of enzyme-linked amplified PDT and PD-L1 inhibition (G6) was associated with superior contralateral tumor suppression compared to individual treatments, highlighting the enhanced efficacy of the synergistic approach [50].

Flow cytometry analyses of contralateral tumors revealed enhanced M1 macrophage polarization, increased DC maturation, and elevated CD8^+^ T cell levels, indicating systemic immune activation (Fig. 8f–h and Fig. S34). The G2 group exhibited significantly higher proportions of M1 macrophages, mature DCs, and CD8^+^ T cells compared to the G1 group. Groups G3 through G5 showed progressive increases in these immune parameters, with the G6 group exhibiting the highest frequencies of M1 macrophages (50.5 %), mature DCs (40.4 %), and CD8^+^ T cells (44.1 %). These findings highlight the robust systemic immune response induced by the treatment, capable of activating antitumor immunity even in distal tumor sites.

In the lung metastasis model, constructed via the tail vein injection of luciferase-labeled SCC7 cells, strong lung metastasis signals were detected in groups G1 to G3 (Fig. 8i). Groups G4 and G5 exhibited a notable reduction in lung metastasis signals, reflecting the inhibitory effect of enzyme-linked amplified PDT. The G6 group, displayed no detectable lung metastasis, demonstrating the most potent suppression of metastatic spread. This enhanced inhibitory effect highlights the efficacy of this synergistic treatment approach as a means of curbing lung metastasis. H&E staining corroborated these findings, with significantly fewer metastatic nodules observed in nanomaterial-treated groups, particularly the G6 group (Fig. 8j).

These results indicate that the combination of enhanced PDT and immunotherapy not only suppresses primary tumor growth but also induces systemic immune responses, preventing the spread of secondary tumors and lung metastasis.

### 3.16 Enhanced PDT-induced transcriptional reprogramming

To further explore the underlying mechanisms of enhanced PDT, we performed a comparative transcriptomic analysis between the PBS group and the HSA/CAT/LOX@IR808+Laser group *in vivo*. This analysis identified 1104 differentially expressed genes (Fig. S35a and b), reflecting significant transcriptional shifts driven by this treatment. Pathway enrichment analysis revealed the strong activation of immune-related pathways, particularly those associated with T cell-mediated immunity and antigen presentation. Key pathways included T cell receptor signaling, Th1/Th2/Th17 cell differentiation, MHC class II protein complexes, and cytokine-cytokine receptor interactions (Fig. S35c–f). Notably, the enrichment of immunological synapse formation and antigen processing pathways further underscored the robust immune activation. Immune cell infiltration analyses confirmed increased proportions of activated immune cells, such as M1 macrophages in the experimental group as opposed to the control group (Fig. S35g). Additionally, gene set variation analysis (GSVA) validated the upregulation of immune-related gene sets (Fig. S35h), further supporting the enhancement of immune activation.

### 3.17 Biocompatibility of the protein vesicle

The safety profile of the protein vesicle was evaluated. The hemolysis assay results showed no hemolysis at nanoparticle concentrations ranging from 50 to 400 μg/mL, indicating that the protein vesicle do not cause damage to red blood cells (Fig. S36). Histological studies of the main organs (heart, liver, spleen, lungs, and kidneys) from all six groups of mice revealed no significant tissue damage, further confirming the safety of the protein vesicle (Fig. S37). Additionally, hematological parameters and biochemical parameters in all groups were within normal ranges (Fig. S38). These comprehensive results demonstrate that the protein vesicle are biocompatible and safe for *in vivo* use. Their non-toxicity and lack of organ damage support their potential application in the context of HNSCC clinical therapy, providing therapeutic benefits without adverse side effects.

## 4 Conclusion

This study introduces a carrier-free flexible dual-enzyme protein vesicle designed to reprogram the hypoxic and lactate-rich TME, enhancing PDT efficacy and immune activation in HNSCC. The carrier-free flexible HSA-based structure ensures superior biocompatibility and tumor-targeting ability. By integrating CAT and LOX, the vesicle effectively alleviates hypoxia, reduces lactate accumulation, and amplifies ROS production through the synergistic action of dual enzymes. This enzyme-driven mechanism significantly enhances PDT efficacy and triggers robust antitumor immunity, marked by M1 macrophage polarization, DC maturation, and increased CD8^+^ T cell infiltration. Moreover, combining optimized PDT with ICB therapy demonstrated synergistic effects, achieving superior tumor suppression, prolonged survival, and reduced metastasis. These findings highlight the collaborative mechanisms of both enzyme-driven amplification and PDT-immune therapy synergy, underscoring the therapeutic potential of this protein vesicle as a novel and effective treatment for HNSCC, warranting further preclinical and clinical exploration.

## CRediT authorship contribution statement

**Yan Zhou:** Writing – original draft, Methodology, Investigation, Formal analysis, Data curation, Conceptualization. **Xiaoquan Xu:** Methodology, Funding acquisition, Data curation, Conceptualization. **Ziyue Zu:** Writing – original draft, Investigation, Data curation, Conceptualization. **Shangyu Lu:** Resources, Methodology, Data curation. **Wei Lu:** Methodology, Investigation. **Xi Luo:** Methodology, Data curation. **Nan Zhong:** Methodology, Investigation. **Yang Liu:** Methodology, Data curation. **Zhaogang Teng:** Methodology, Investigation. **Shouju Wang:** Writing – review & editing, Supervision, Resources, Conceptualization. **Feiyun Wu:** Writing – review & editing, Supervision, Resources, Funding acquisition, Conceptualization.

## Declaration of competing interest

The authors declare that they have no known competing financial interests or personal relationships that could have appeared to influence the work reported in this paper.

## Data Availability

The datasets used during the current study are available from the corresponding author on reasonable request.
