# Supplementary material for: Breaking the siege of hypoxia and lactate: carrier-free flexible dual-enzyme protein vesicle to ignite photodynamic-immune storm in head and neck squamous cell carcinoma
Source: Mater Today Bio. 2025 Jul 12;33:102082. doi: 10.1016/j.mtbio.2025.102082 (PMC12284486; doi:10.1016/j.mtbio.2025.102082)
Supplement: Multimedia component 1 [file mmc1.pdf]

Supporting Information

Table S1. Encapsulation efficiency and loading capacity of individual components in HSA/CAT/LOX@IR808

NO	HSA input (mg)	HSA content (mg)	Total weight (mg)	Encapsulation efficiency (%)	Loading capacity (%)
1	10.0	8.3	15.5	83.0	53.5
2	10.0	7.9	14.9	79.0	53.0
3	10.0	8.0	17.2	80.0	46.5
Average	10.0	8.1	15.9	80.7	51.0

NO	CAT input (mg)	CAT content (mg)	Total weight (mg)	Encapsulation efficiency (%)	Loading capacity (%)
1	3.0	2.5	15.5	83.3	16.1
2	3.0	2.2	14.9	73.3	14.8
3	3.0	2.4	17.2	80.0	14.0
Average	3.0	2.4	15.9	78.9	15.0

NO	LOX input (mg)	LOX content (mg)	Total weight (mg)	Encapsulation efficiency (%)	Loading capacity (%)
1	2.0	1.3	15.5	65.0	8.4
2	2.0	1.3	14.9	65.0	8.7
3	2.0	1.4	17.2	70.0	8.1
Average	2.0	1.3	15.9	66.7	8.4

NO	IR808 input (mg)	IR808 content (mg)	Total weight (mg)	Encapsulation efficiency (%)	Loading capacity (%)
1	2.0	1.2	15.5	60.0	7.7
2	2.0	1.3	14.9	65.0	8.7
3	2.0	1.4	17.2	70.0	8.1
Average	2.0	1.3	15.9	65.0	8.2

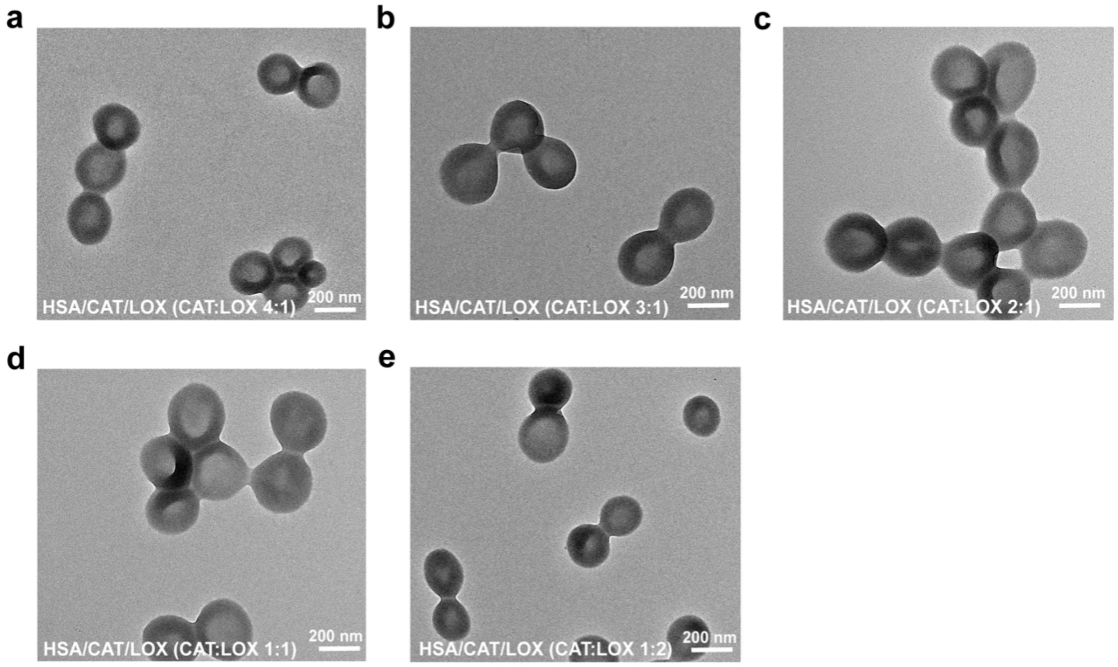

Fig. S1. Representative transmission electron microscopy (TEM) images of HSA/CAT/LOX with different CAT:LOX ratios: (a) 4:1, (b) 3:1, (c) 2:1, (d) 1:1, and (e) 1:2.

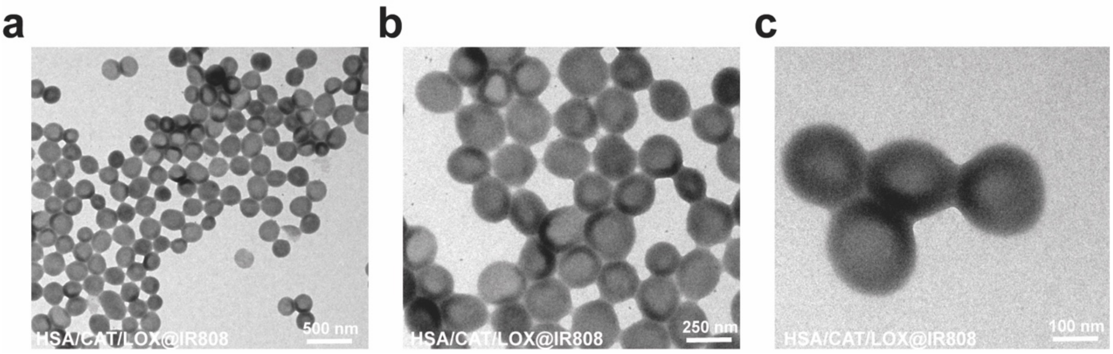

Fig. S2. TEM images of HSA/CAT/LOX@IR808 showing stable hollow vesicle morphology after IR808 conjugation.

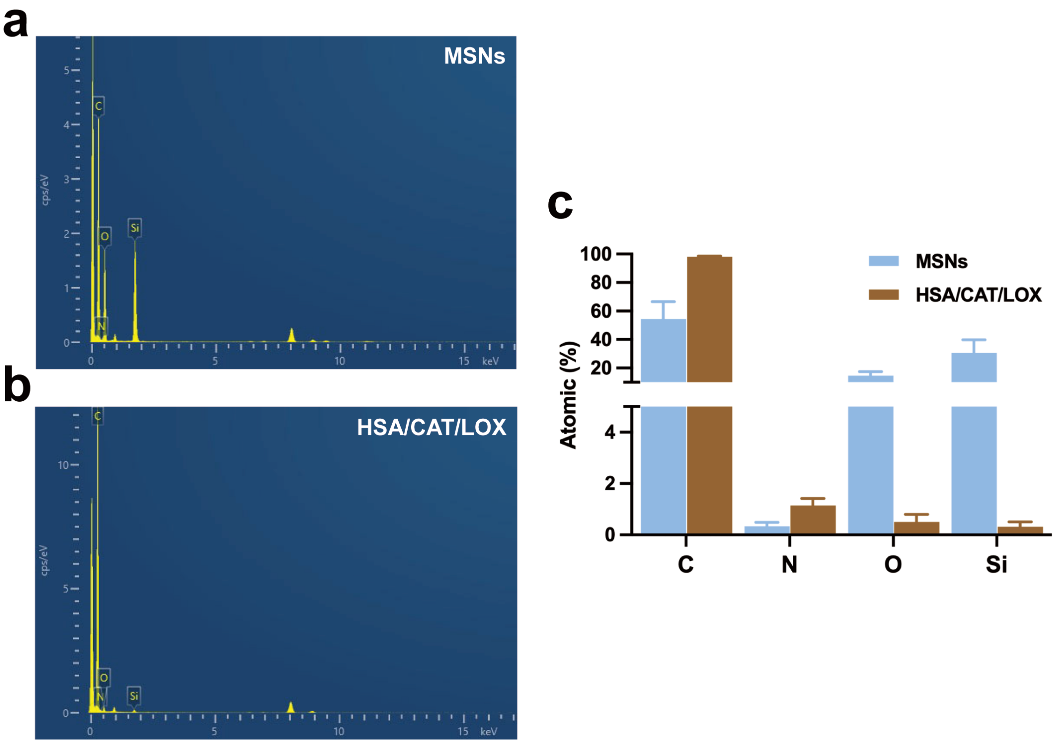

Fig S3. Quantitative Energy Dispersive X-ray Spectroscopy (EDS) analysis of (a) MSNs, (b) HSA/CAT/LOX, and (c) bar chart showing decreased O and Si content after template removal.

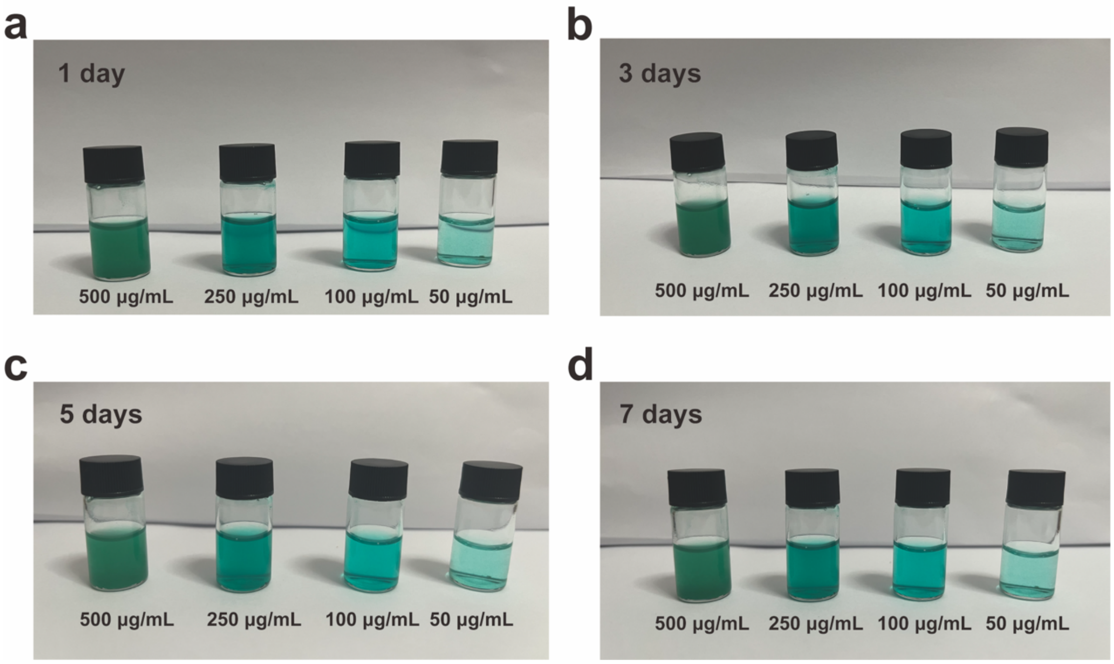

Fig. S4. Photographs showing good solubility and stability of HSA/CAT/LOX@IR808 dispersed in PBS buffer (pH 7.4). The solution remained clear and homogeneous without visible aggregation for 7 days.

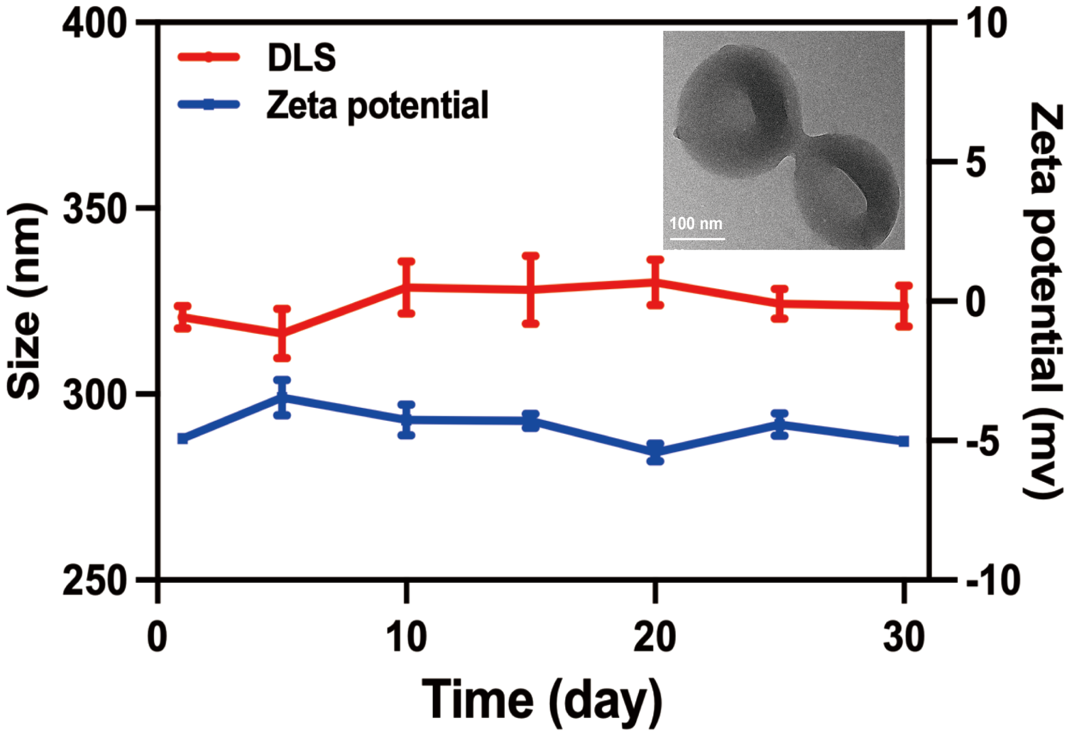

Fig. S5. Stability evaluation of HSA/CAT/LOX@IR808 over time. TEM images show that the vesicles retained their hollow morphology after 30 days of storage. Dynamic light scattering (DLS) and Zeta potential measurements at different time points further confirmed the stability.

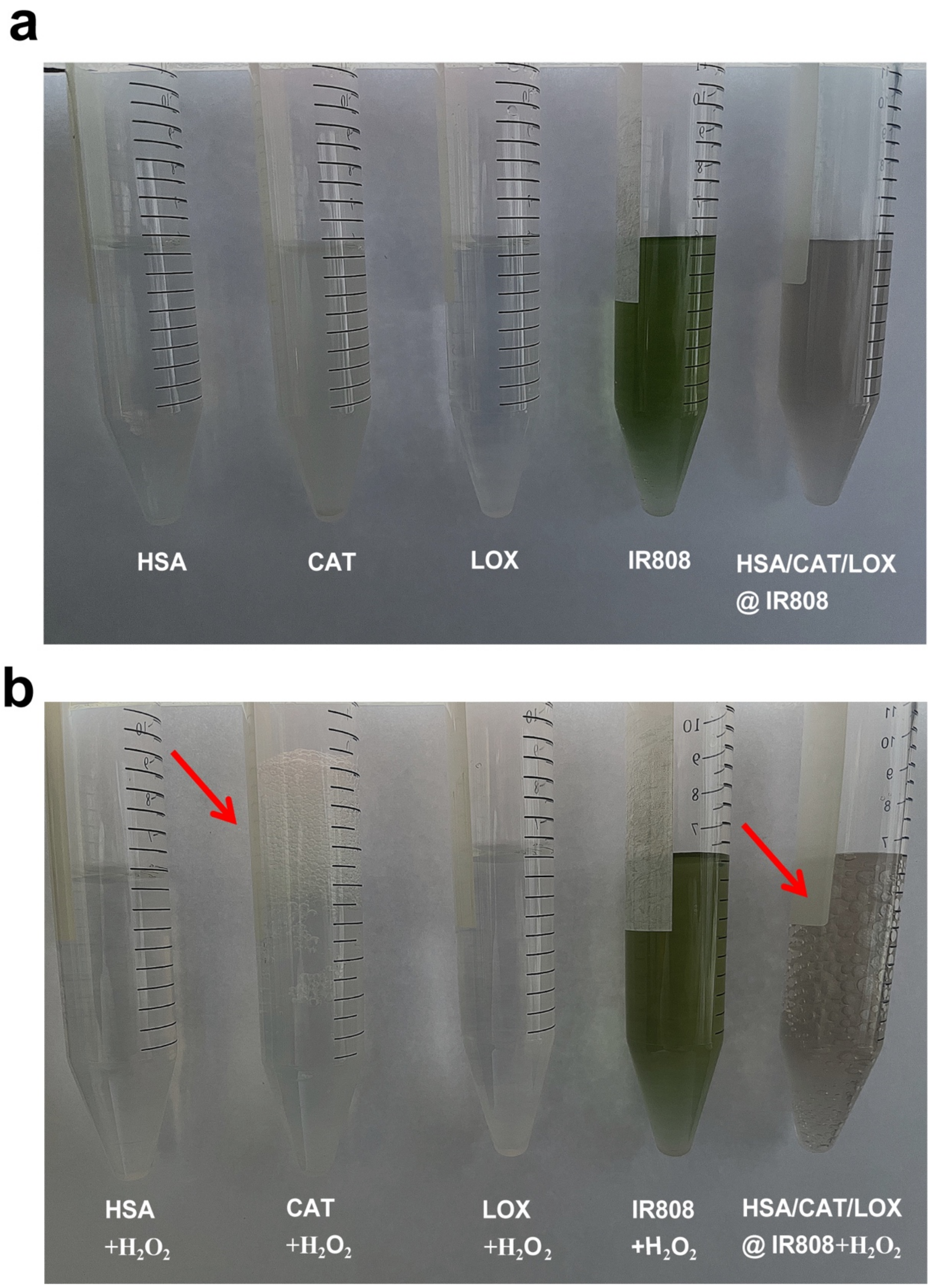

Fig. S6. Bubble formation by different materials with or without H_2O_2. (a) Samples without H_2O_2 showed no bubble formation. (b) Both CAT and HSA/CAT/LOX@IR808 generate substantial bubbles in the presence of H_2O_2, indicating that CAT activity in HSA/CAT/LOX@IR808 remains effective.

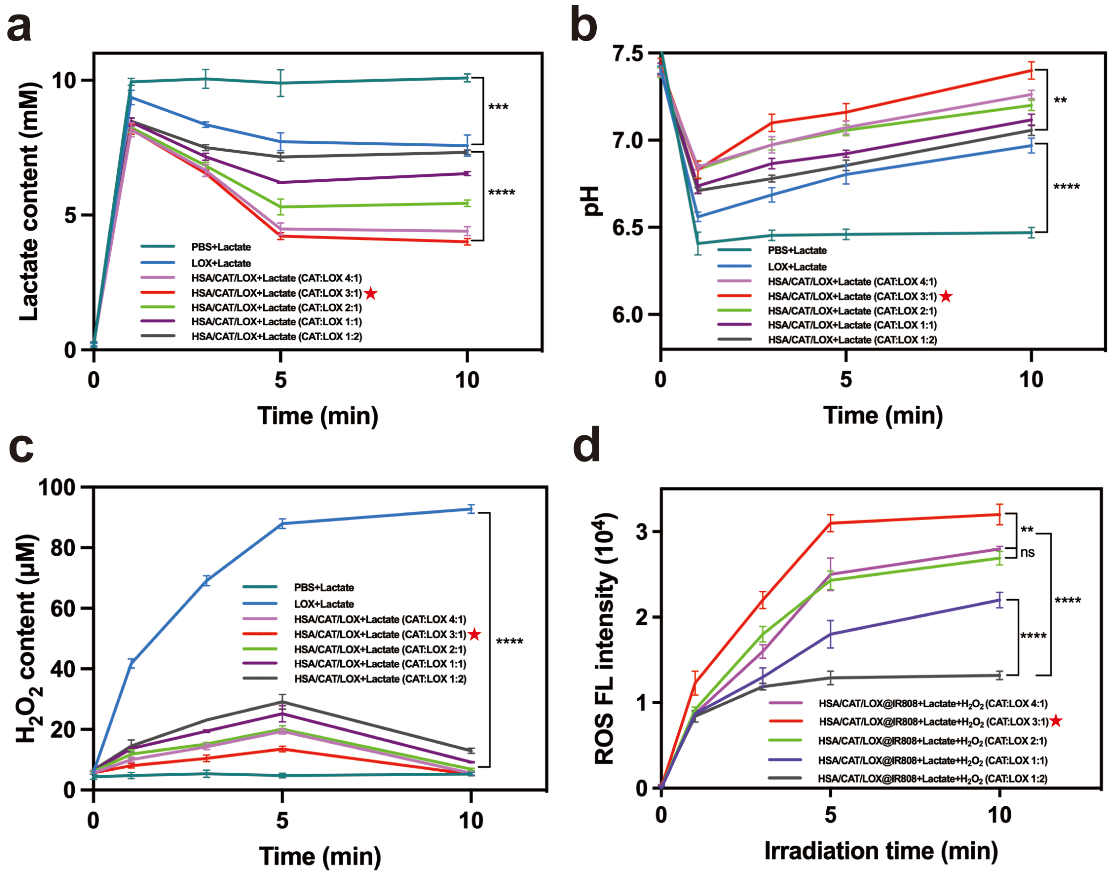

Fig. S7. Enzyme activity and ROS generation at different CAT-LOX ratios. (a-c) Changes in lactate consumption, pH increase, and H_2O_2 depletion across different CAT-LOX ratios. The 3:1 ratio showed the most significant effects. (d) ROS generation ($^1O_2$) under NIR irradiation, with the 3:1 CAT-LOX ratio producing the highest ROS levels. Data are presented as mean ± SD. Statistical analyses were performed using one-way ANOVA with Tukey's post hoc test.

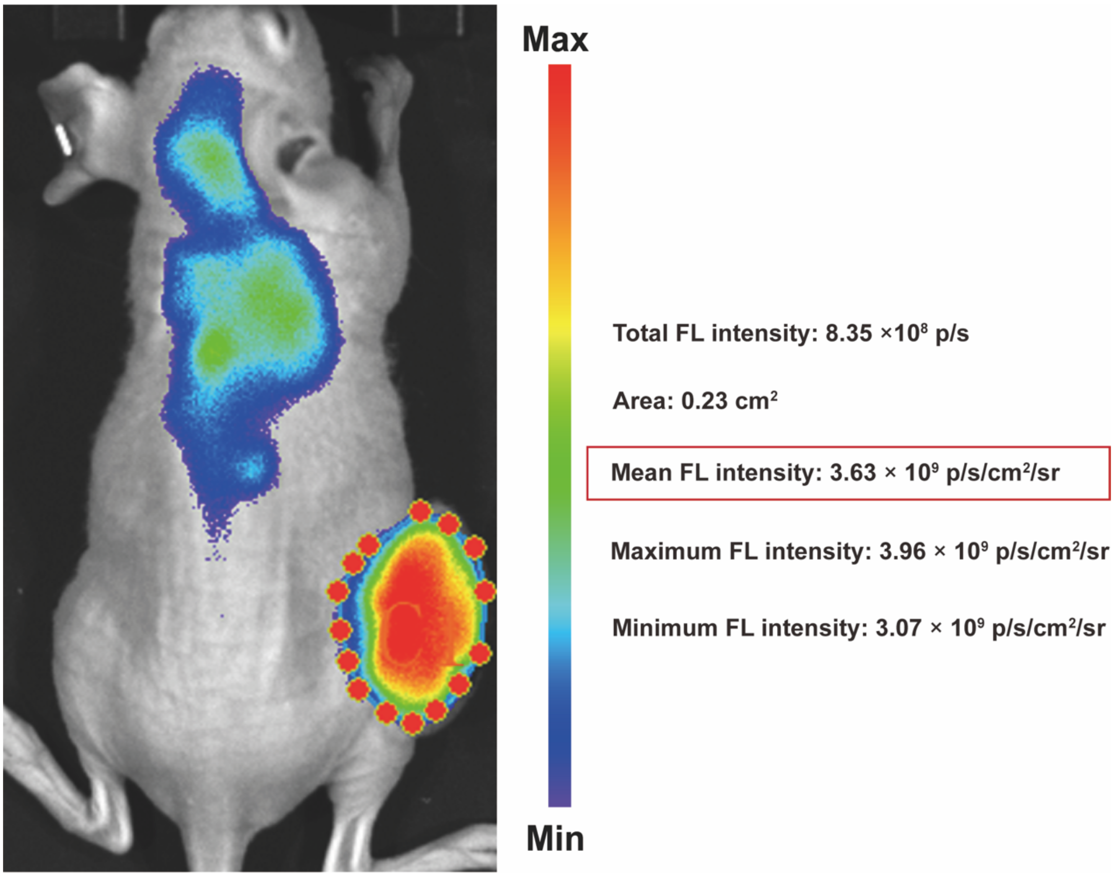

Fig. S8. Representative illustration of tumor fluorescence (FL) quantification using Aniview. The software provides total, average, maximum, and minimum FL intensity within the ROI. Average intensity was used for comparative analysis across group.

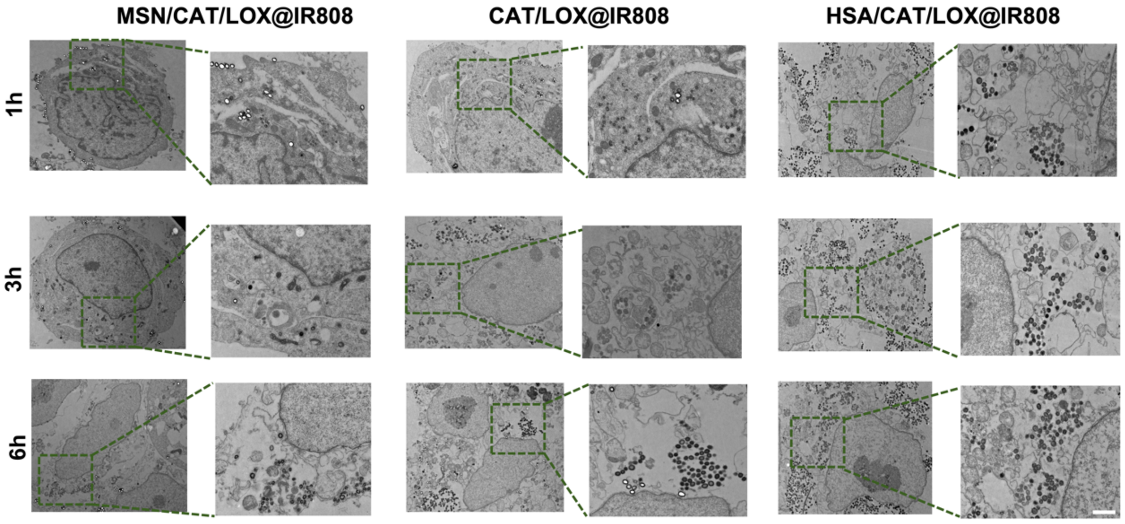

Fig. S9. Biological electron microscopy images showing the uptake of MSN/CAT/LOX@IR808, CAT/LOX@IR808, and HSA/CAT/LOX@IR808 in SCC7 cells at 1h, 3h, and 6h. HSA/CAT/LOX@IR808 shows the highest internalization efficiency, followed by CAT/LOX@IR808 and MSN/CAT/LOX@IR808, highlighting the roles of flexibility and HSA in enhancing cellular uptake. Scale bar: 1 μm.

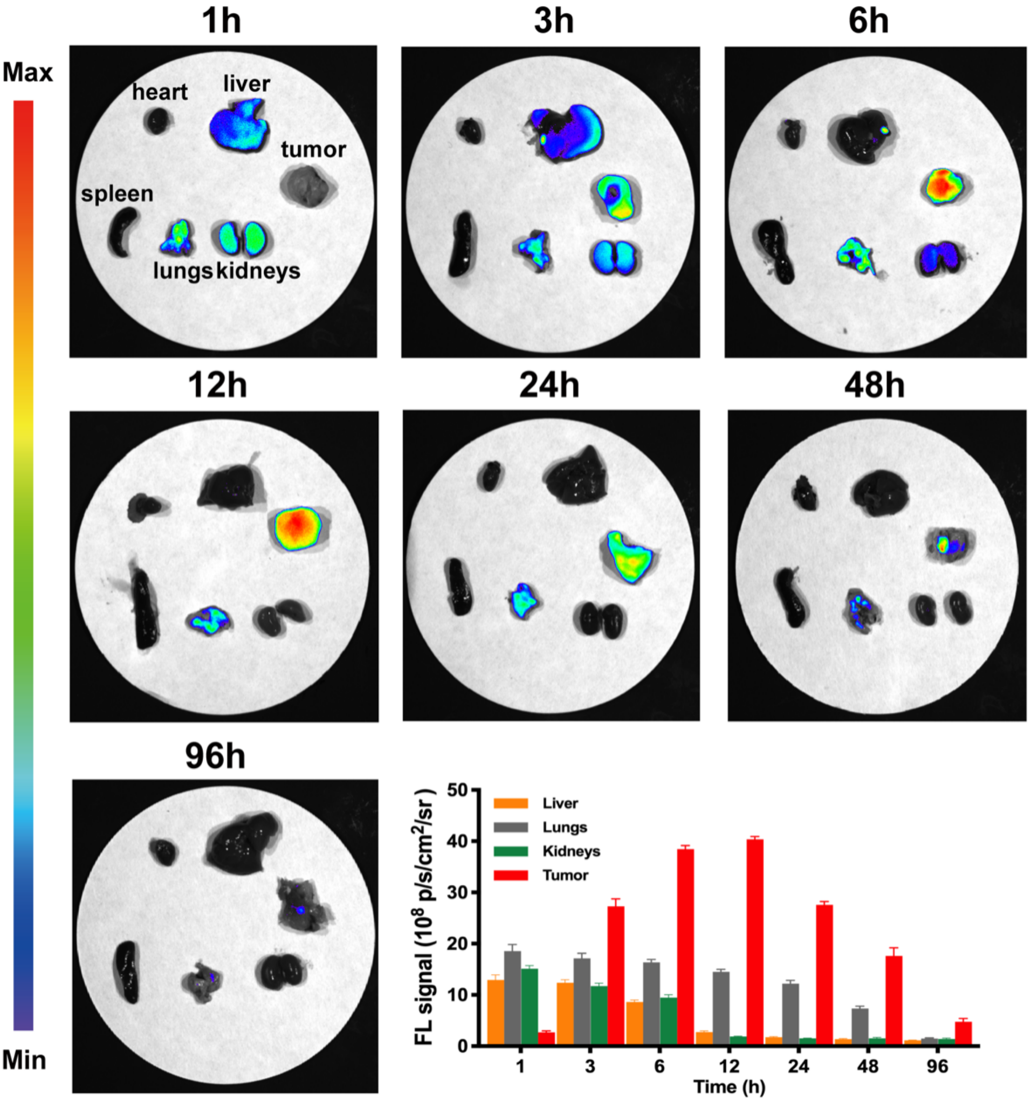

Fig. S10. The FL distributions of HSA/CAT/LOX@IR808 in major organs (liver, kidneys, and lungs) and tumors over time.

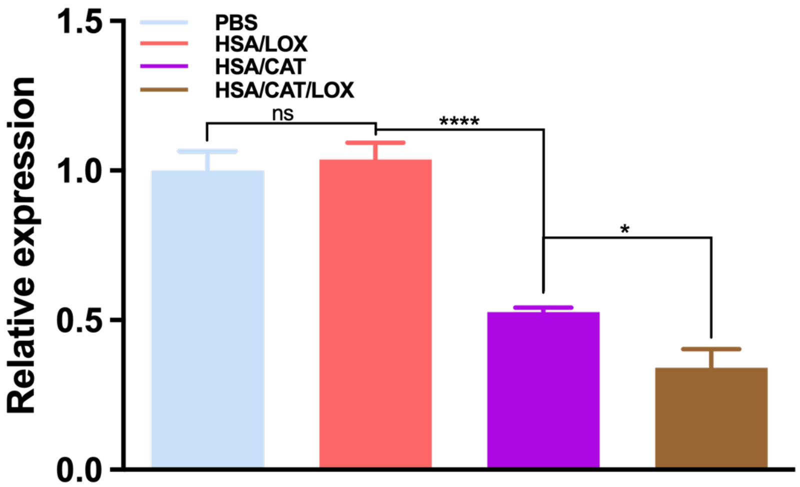

Fig. S11. Quantitative results of HIF-1α western blotting (WB) analysis for different treatment groups. Data are presented as mean ± SD. Statistical analyses were performed using one-way ANOVA with Tukey's post hoc tests.

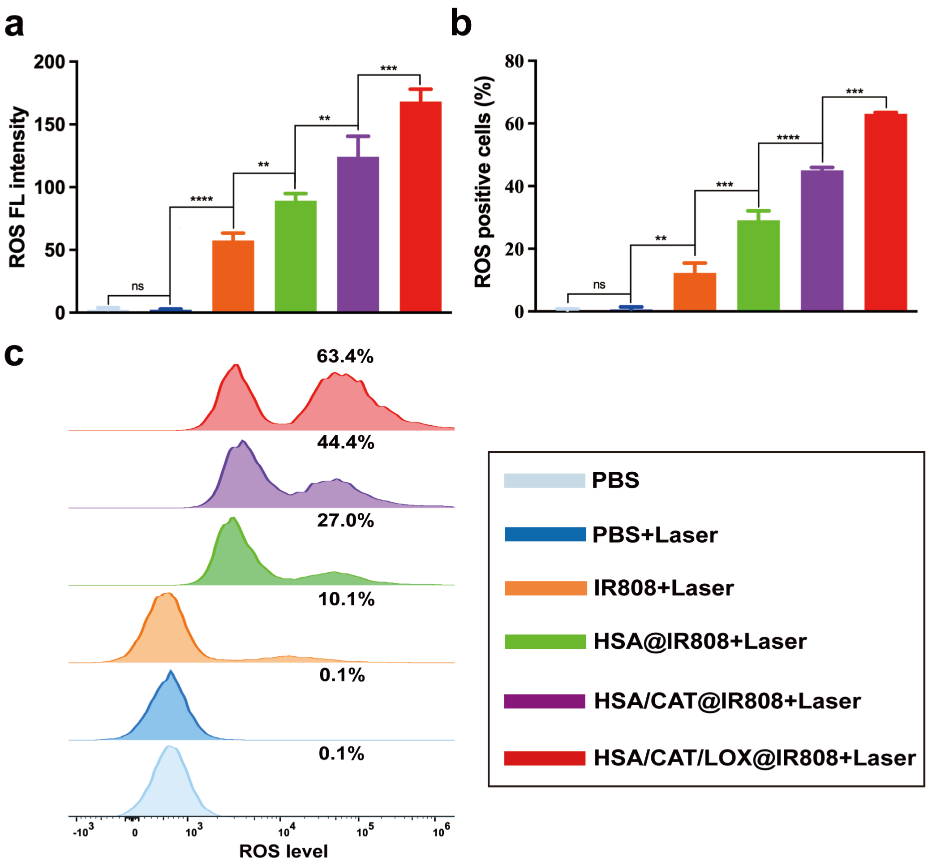

Fig. S12. ROS generation in SCC7 cells. (a) Quantitative results from FL microscopy. (b, c) Flow cytometry analysis of ROS generation following different treatment. Data are presented as mean ± SD. Statistical analyses were performed using one-way ANOVA with Tukey's post hoc tests.

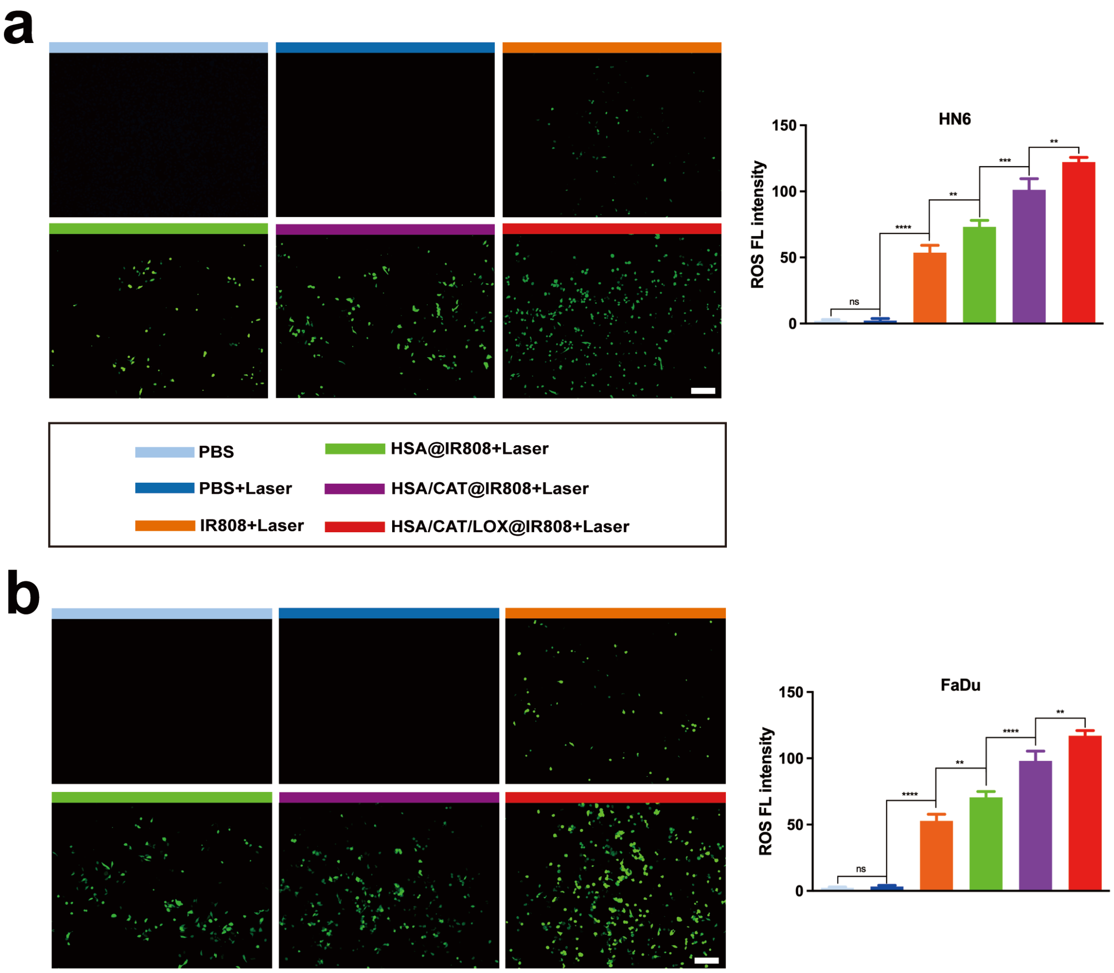

Fig. S13. ROS generation in HN6 and FaDu cells. (a) ROS generation in HN6 cells treated with different formulations under 808 nm laser irradiation. (b) ROS generation in FaDu cells under the same conditions. Both panels show increased ROS levels with enzyme components, peaking in the HSA/CAT/LOX@IR808+Laser group. Scale bar: 50 μm. Data are presented as mean ± SD. Statistical analyses were performed using one-way ANOVA with Tukey's post hoc test.

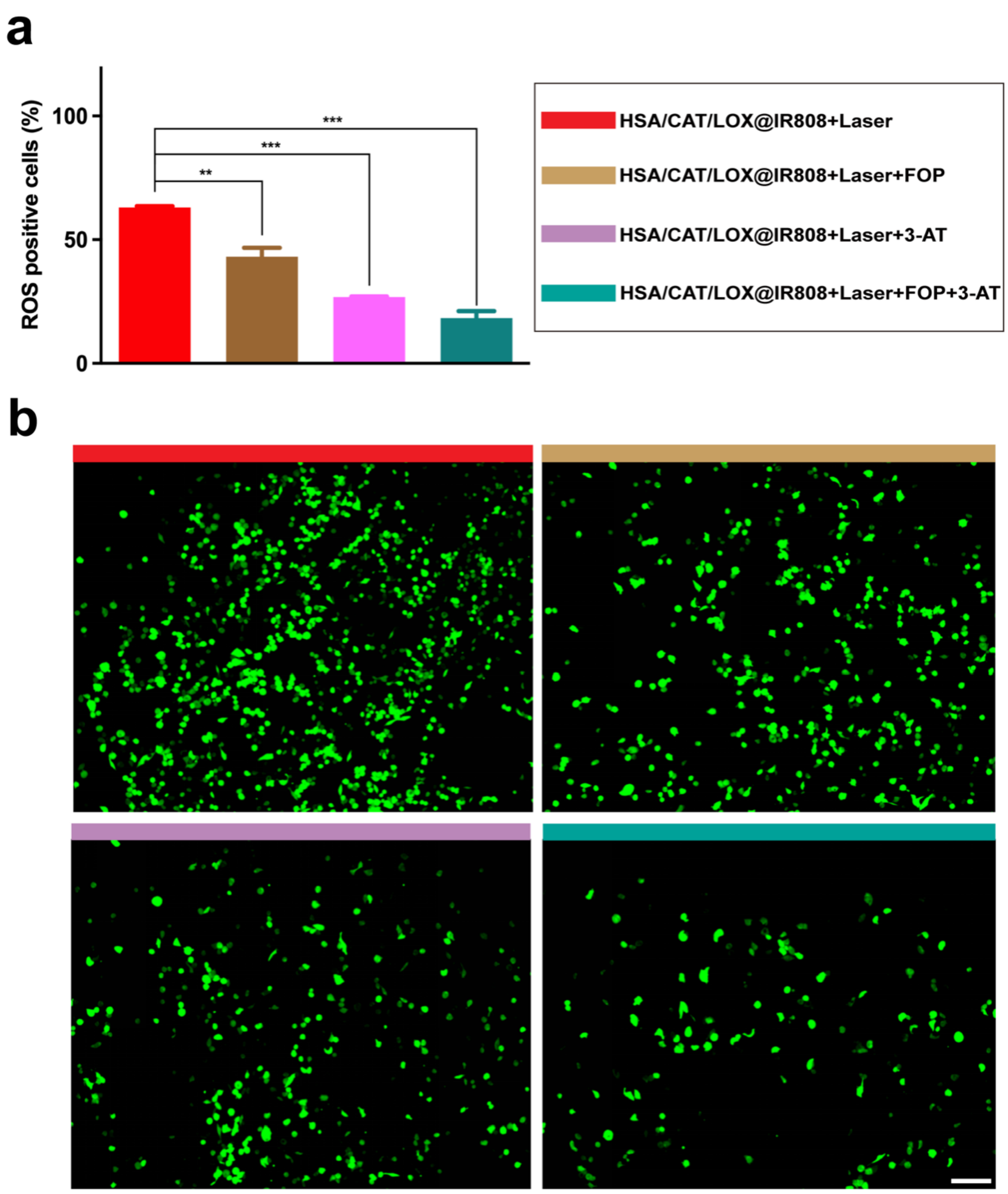

Fig. S14. ROS amplification through enzyme cascade and effects of LOX and CAT inhibition. (a) Flow cytometry quantification of ROS levels. (b) FL microscopy analysis showing ROS signal after treatment with LOX inhibitor FOP, CAT inhibitor 3-AT, and the combination of FOP + 3-AT. Scale bar: 50 μm. Data are presented as mean ± SD. Statistical analyses were performed using one-way ANOVA followed by Tukey's post hoc tests.

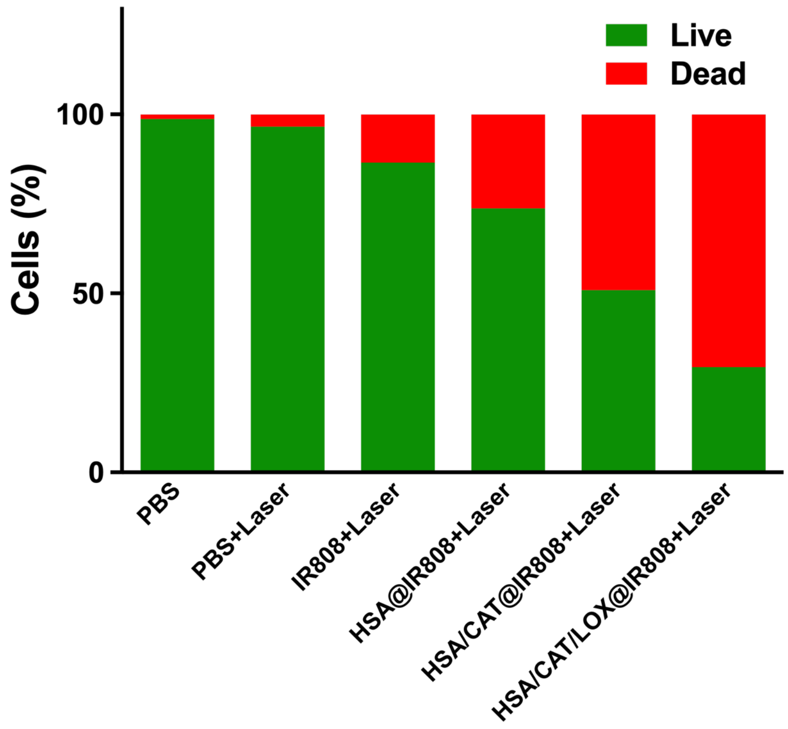

Fig. S15. Quantitative FL staining of SCC7 cells with different treatments, showing increased cell death in the HSA/CAT/LOX@IR808+Laser group, consistent with enhanced photodynamic therapy (PDT) efficacy.

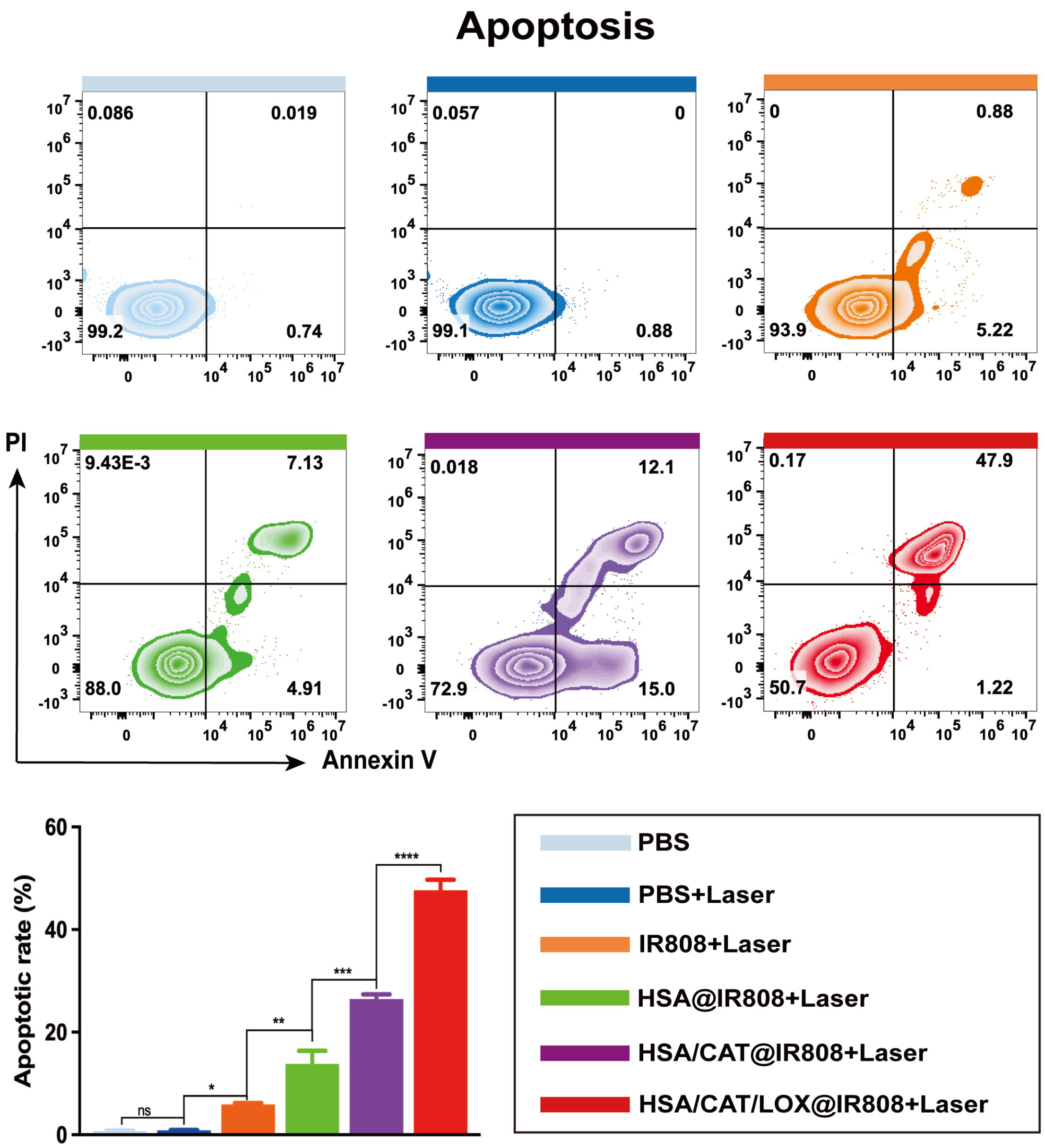

Fig. S16. Flow cytometry analysis of apoptosis after PDT treatment. Flow cytometry results showing the apoptotic effects of different treatment groups. The HSA/CAT/LOX@IR808+Laser group exhibited the highest apoptosis rate, attributed to enhanced ROS production and activation of intrinsic apoptotic pathways. Data are presented as mean ± SD. Statistical analyses were performed using one-way ANOVA with Tukey's post hoc tests.

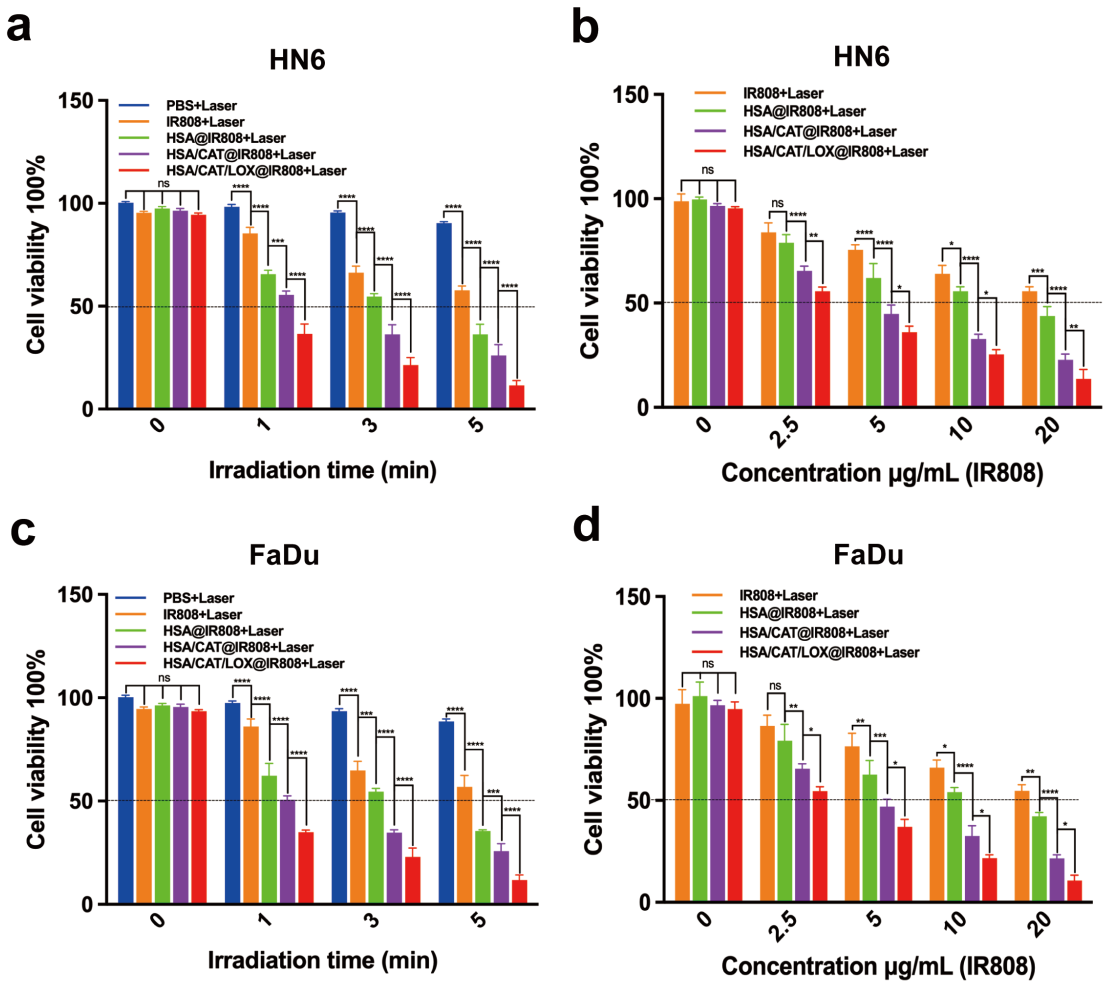

Fig. S17. CCK-8 assay results of HSA/CAT/LOX@IR808 in two cell lines. Cell viability of HN6 cells at different time points (a) and IR808 concentrations (b). Cell viability of FaDu cells at different time points (c) and IR808 concentrations (d). Data are presented as mean ± SD. Statistical analyses were performed using one-way ANOVA with Tukey’s post hoc tests.

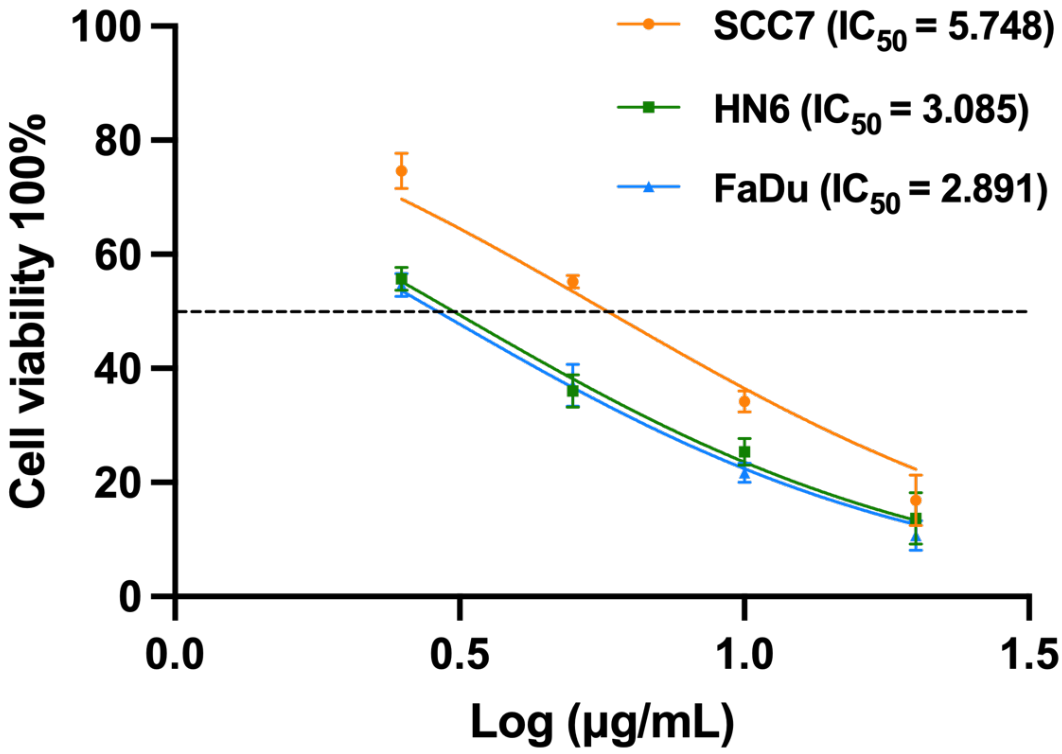

Fig. S18 IC_{50} values of HSA/CAT/LOX@IR808 determined by CCK-8 assays in SCC7, HN6, and FaDu cells.

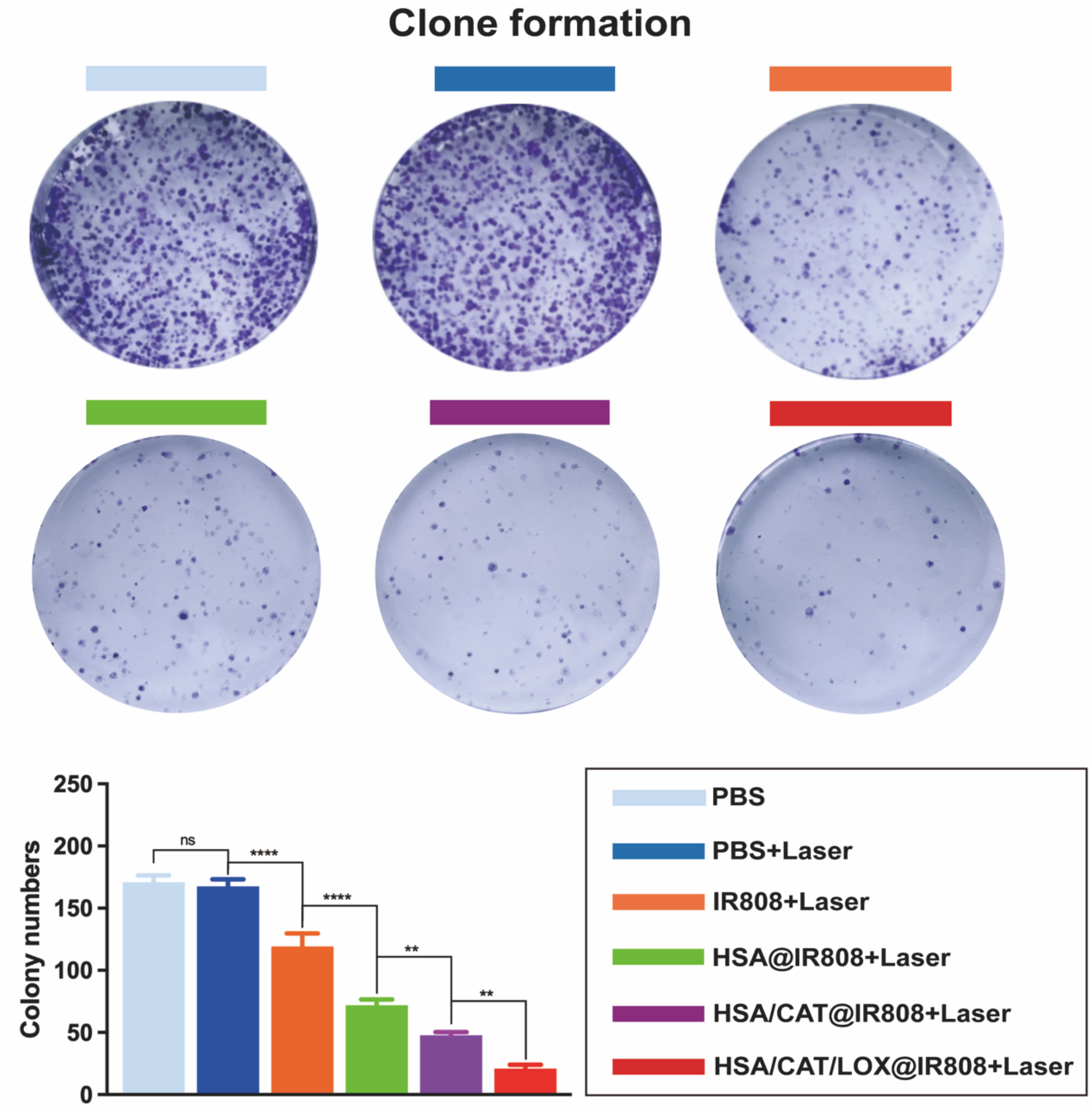

Fig. S19. Colony formation assay. Colony formation images and quantification showing the inhibitory effects of different treatments on tumor cell proliferation. The HSA/CAT/LOX@IR808+Laser group exhibited the most significant reduction in colony numbers, highlighting its superior anti-proliferative effects. Data are presented as mean ± SD. Statistical analyses were performed using one-way ANOVA with Tukey's post hoc tests.

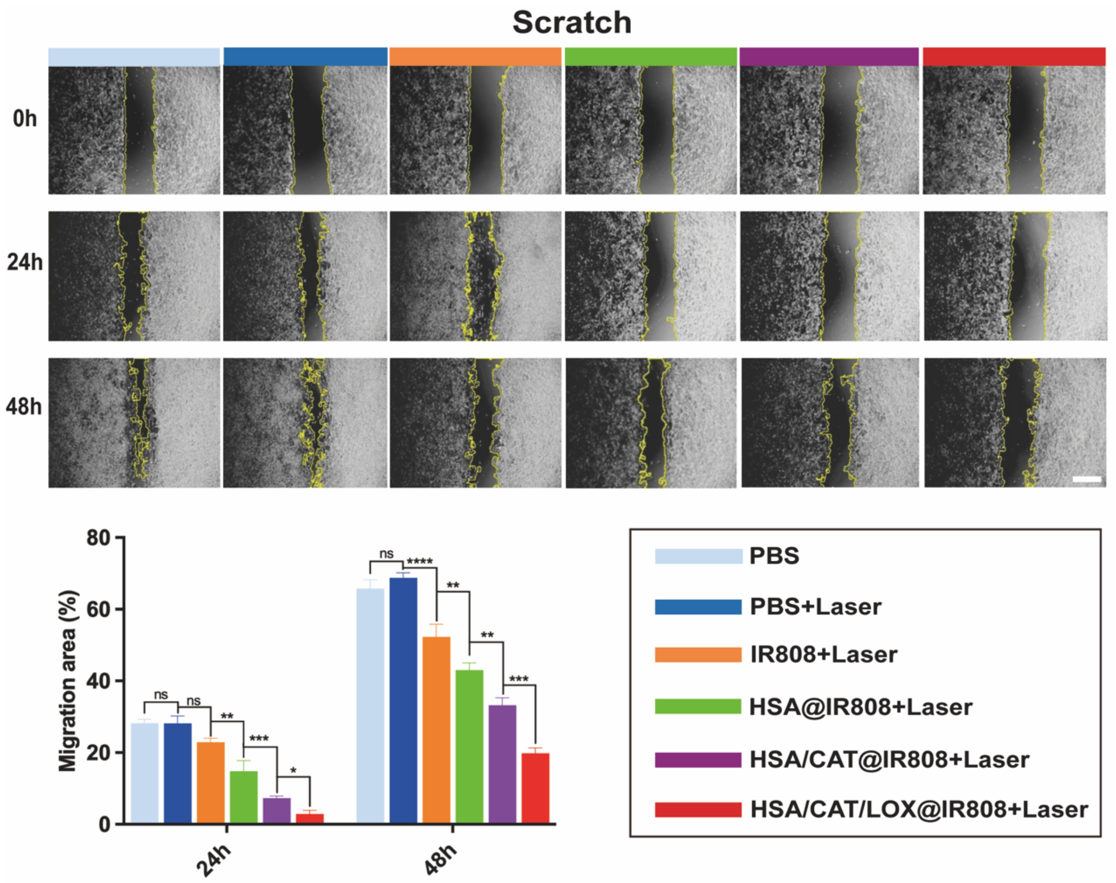

Fig. S20. Wound healing assay after PDT treatment. Representative images and quantification of wound closure at different time points. The enzyme-loaded protein vesicle significantly impaired cell migration, with the HSA/CAT/LOX@IR808+Laser group showing the least wound closure, indicating the strongest inhibition of migratory activity. Scale bar: 200 µm. Data are presented as mean ± SD. Statistical analyses were performed using one-way ANOVA with Tukey's post hoc tests.

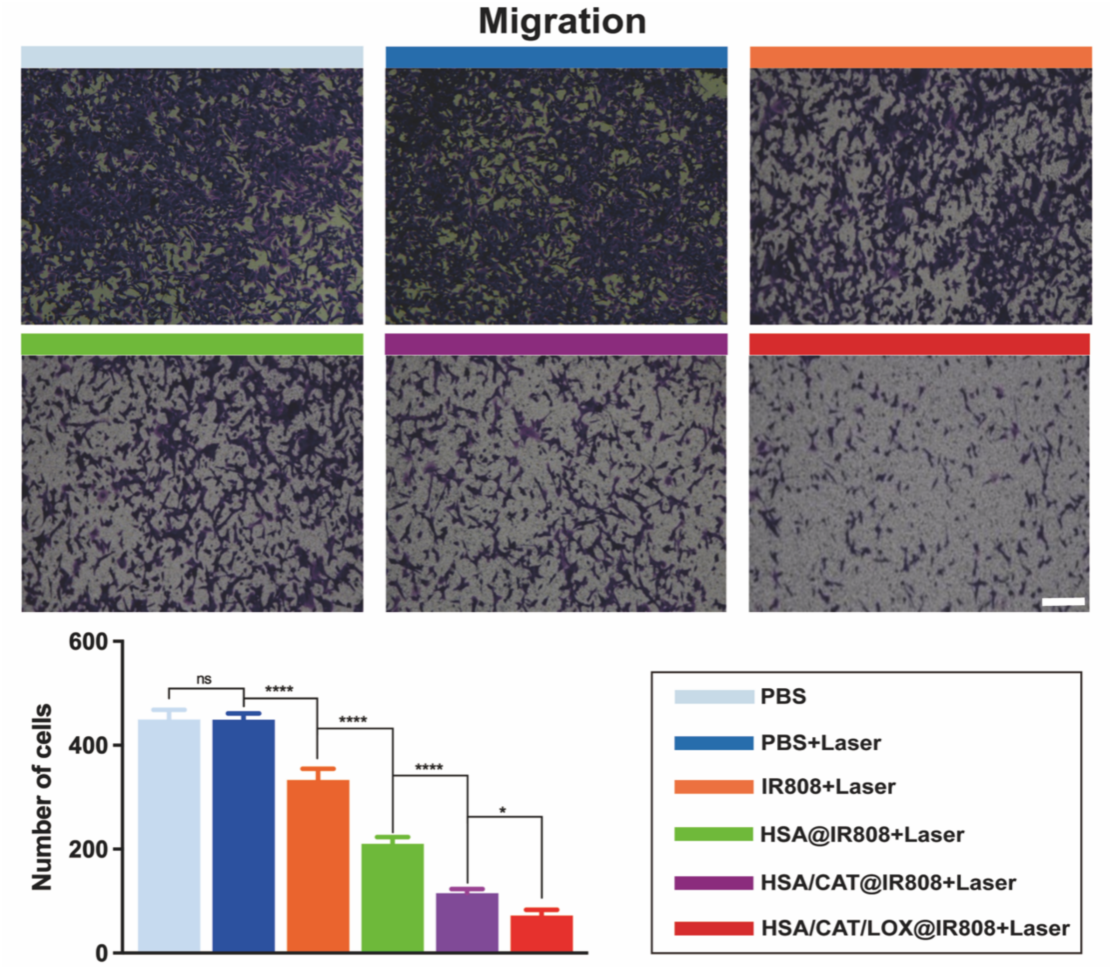

Fig. S21. Transwell migration assay. The results showing the effects of different treatments on cell migration. The enzyme-loaded protein vesicle group significantly reduced cell migration, with the HSA/CAT/LOX@IR808+Laser group exhibiting the greatest inhibition. Scale bar: 100 µm. Data are presented as mean ± SD. Statistical analyses were performed using one-way ANOVA with Tukey's post hoc tests.

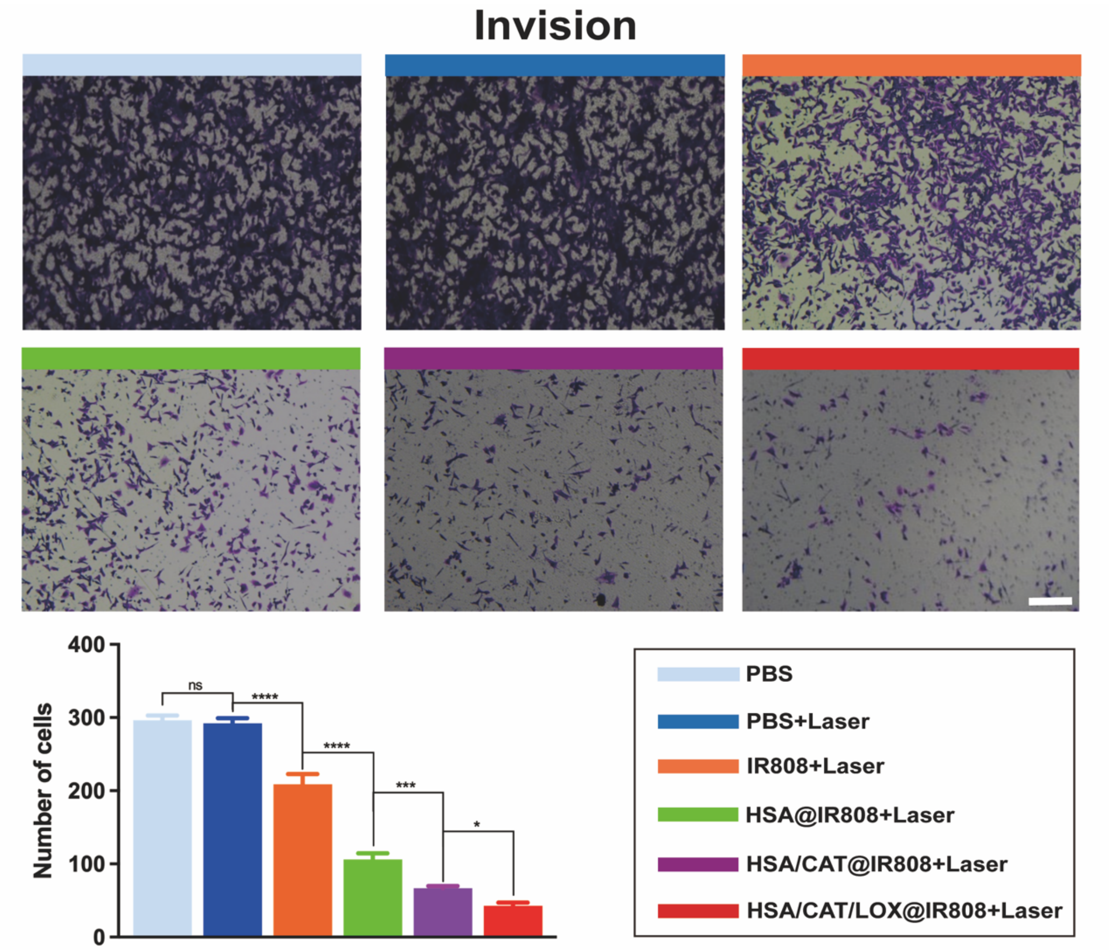

Fig. S22. Transwell invasion assay. The enzyme-loaded protein vesicle group markedly suppressed invasion, with the HSA/CAT/LOX@IR808+Laser group demonstrating the most substantial inhibition, indicating its strong anti-invasive potential. Scale bar: 100 μm. Data are presented as mean ± SD. Statistical analyses were performed using one-way ANOVA with Tukey's post hoc tests.

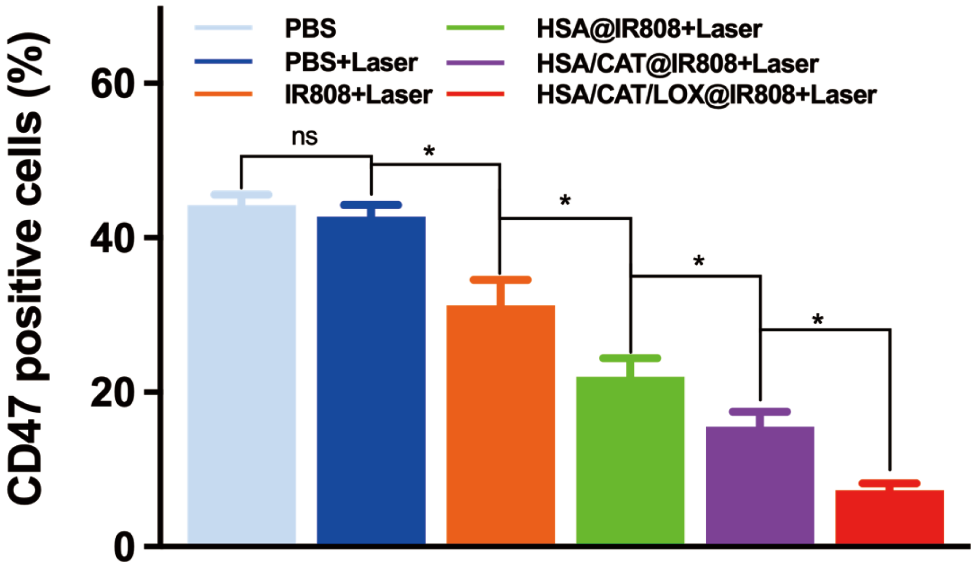

Fig. S23. Quantitative flow cytometry analysis of CD47 expression in SCC7 cells with different post-treatments. Data are presented as mean ± SD. Statistical analyses were performed using one-way ANOVA with Tukey's post hoc tests.

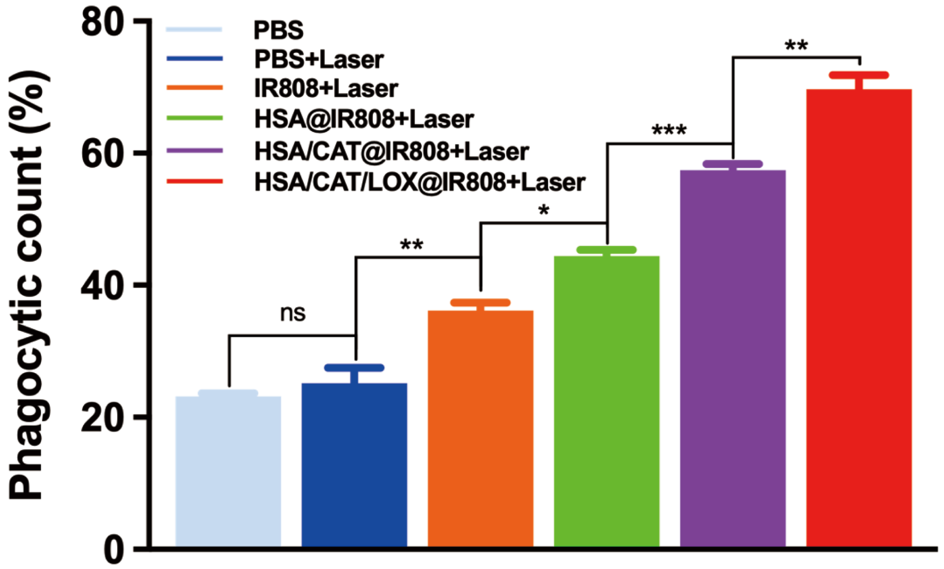

Fig. S24. Quantitative histogram of macrophage phagocytosis index in different treatment groups. Data are presented as mean ± SD. Statistical analyses were performed using one-way ANOVA with Tukey's post hoc tests.

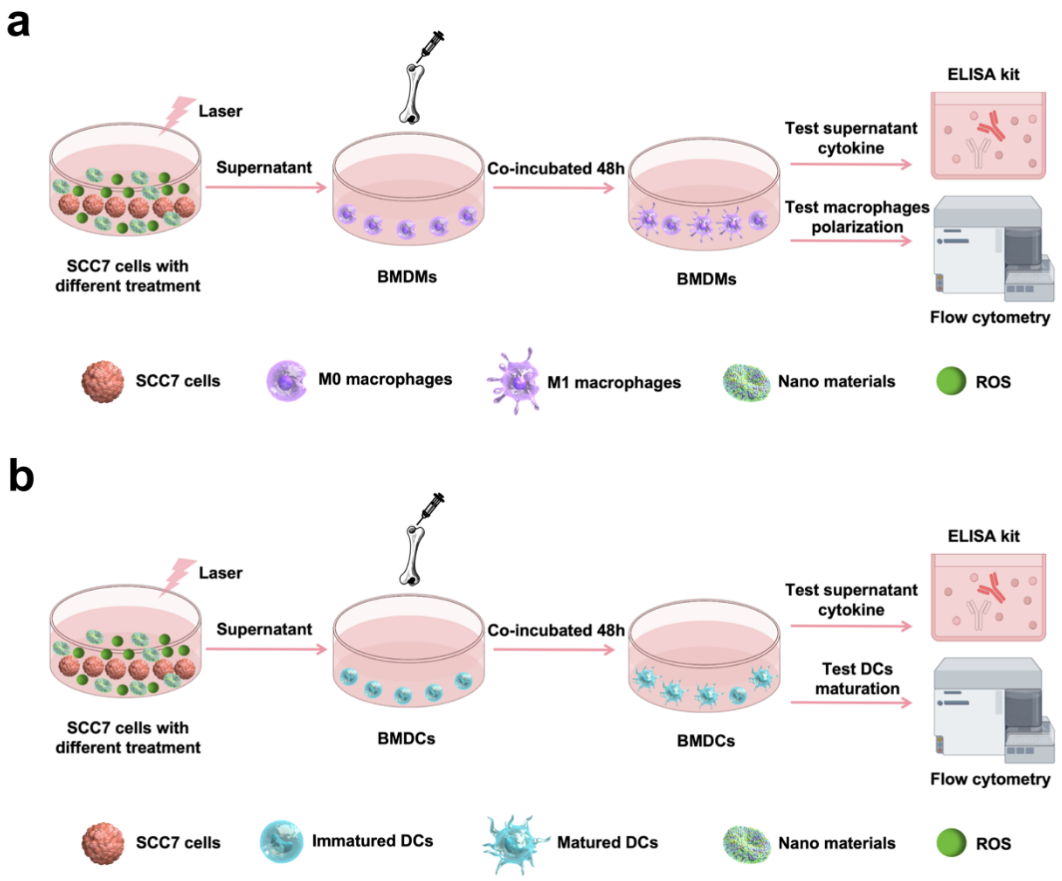

Fig. S25. Schematic diagrams of the co-culture experimental workflow. Supernatants from nanoparticle-treated SCC7 cells were co-incubated with (a) bone marrow-derived macrophages (BMDMs) and (b) bone marrow-derived dendritic cells (BMDCs) for 48 hours, followed by flow cytometry and ELISA to assess immune cell activation and cytokine levels.

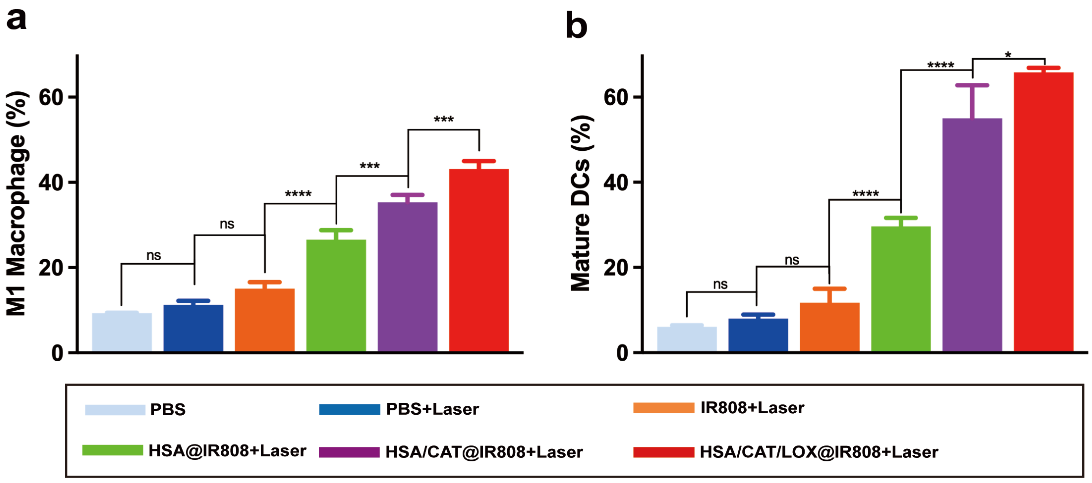

Fig. S26. Quantitative flow cytometry analysis of BMDM M1 polarization (a) and BMDC maturation (b). Data are presented as mean ± SD. Statistical analyses were performed using one-way ANOVA with Tukey's post hoc tests.

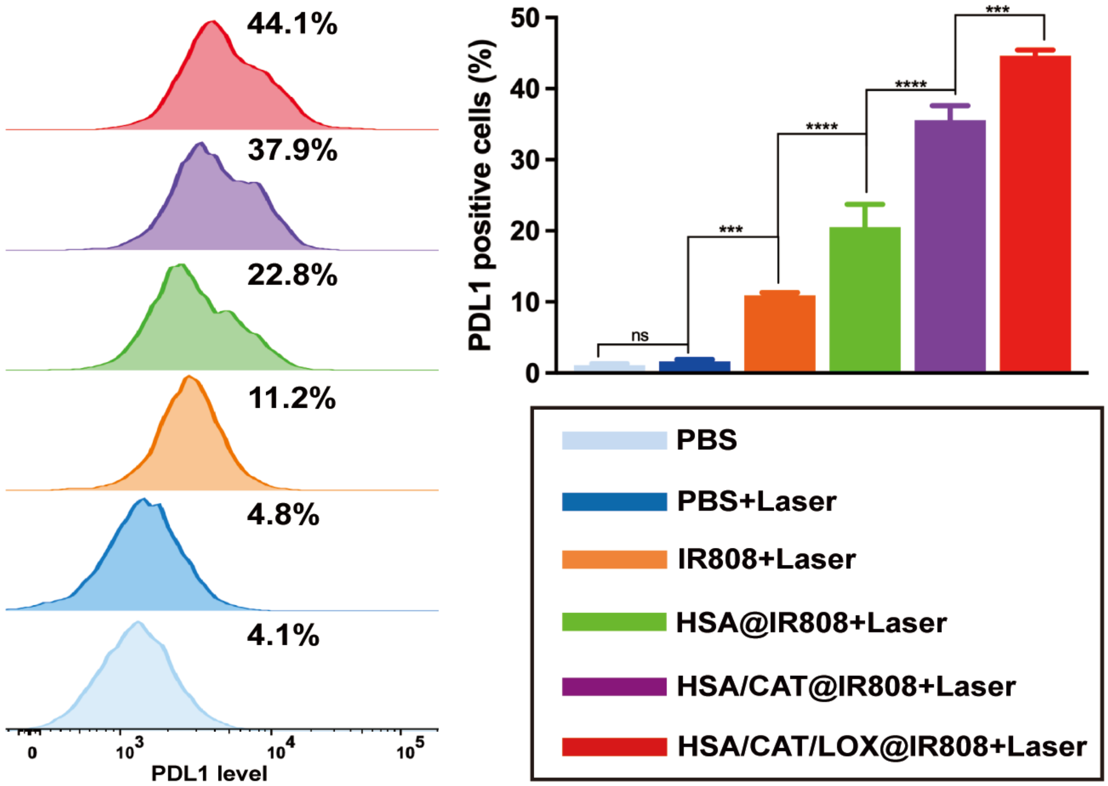

Fig. S27. Flow cytometry analysis of PD-L1 expression in SCC7 cells with different post-treatments. Data are presented as mean ± SD. Statistical analyses were performed using one-way ANOVA with Tukey's post hoc tests.

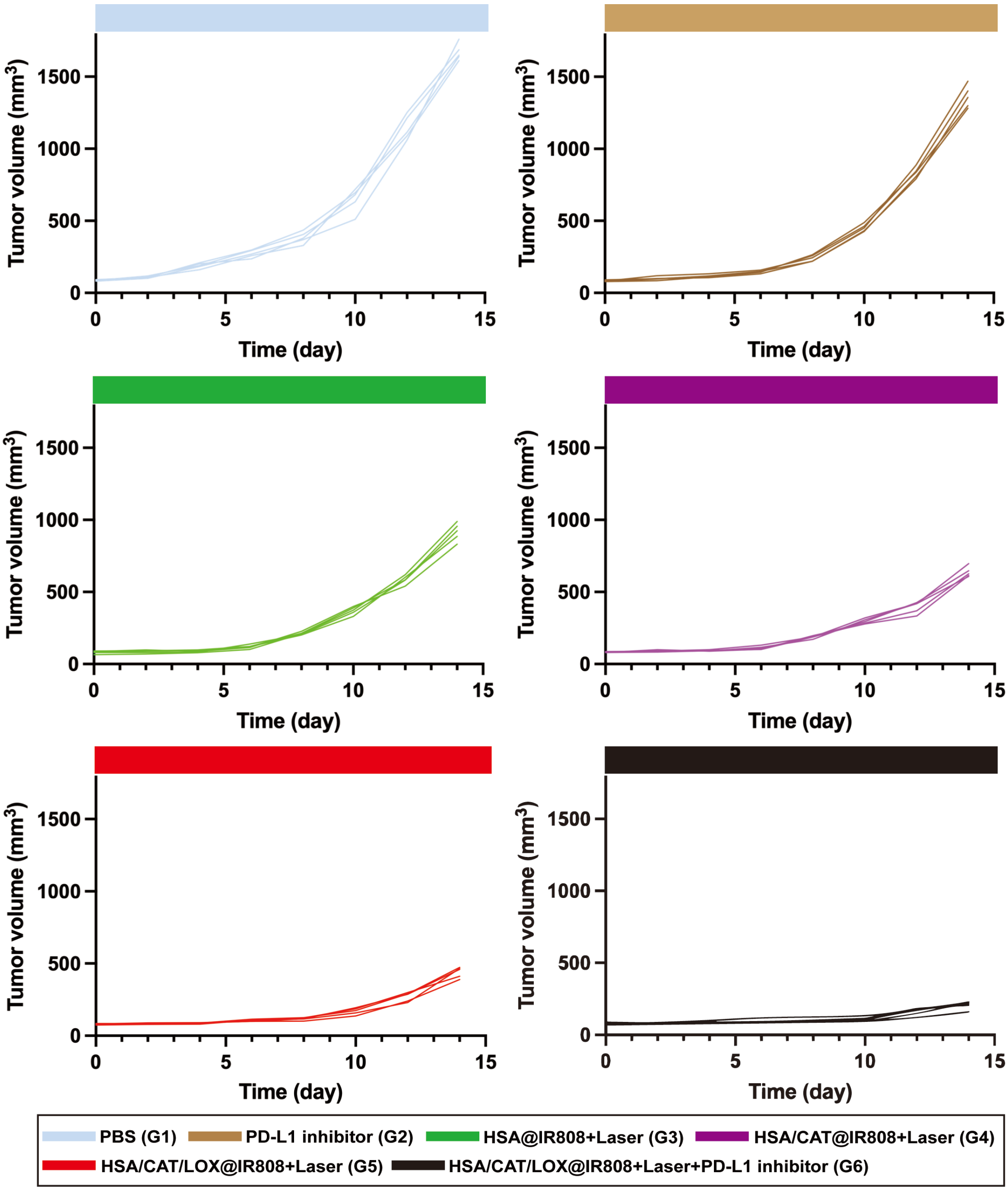

Fig. S28. Tumor volume was monitored for 14 days, showing varying degrees of growth inhibition across treatment groups. The combination therapy (G6) resulted in the most significant suppression, highlighting its enhanced antitumor efficacy.

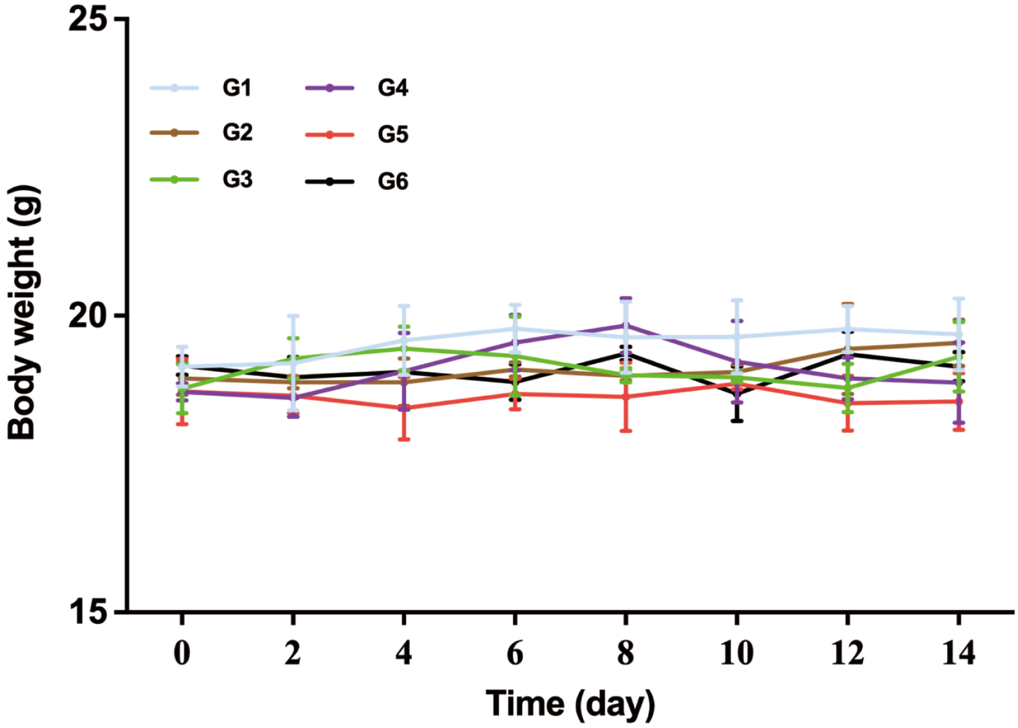

Fig. S29. Body weight changes in SCC7 tumor-bearing mice after administering various treatments. Data are presented as mean ± SD.

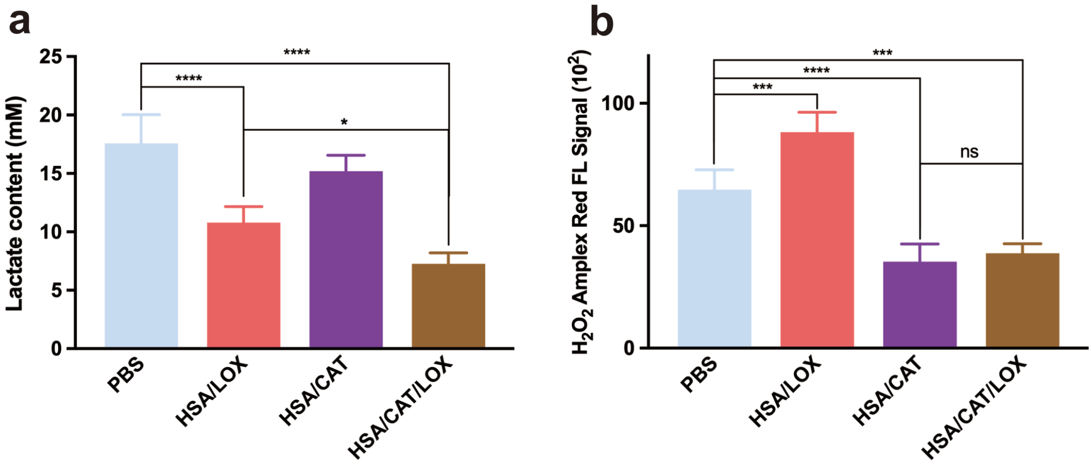

Fig. S30. (a) Intratumoral lactate levels and (b) H_2O_2 levels measured after treatment in different groups. Data are presented as mean ± SD. Statistical analyses were performed using one-way ANOVA with Tukey's post hoc tests.

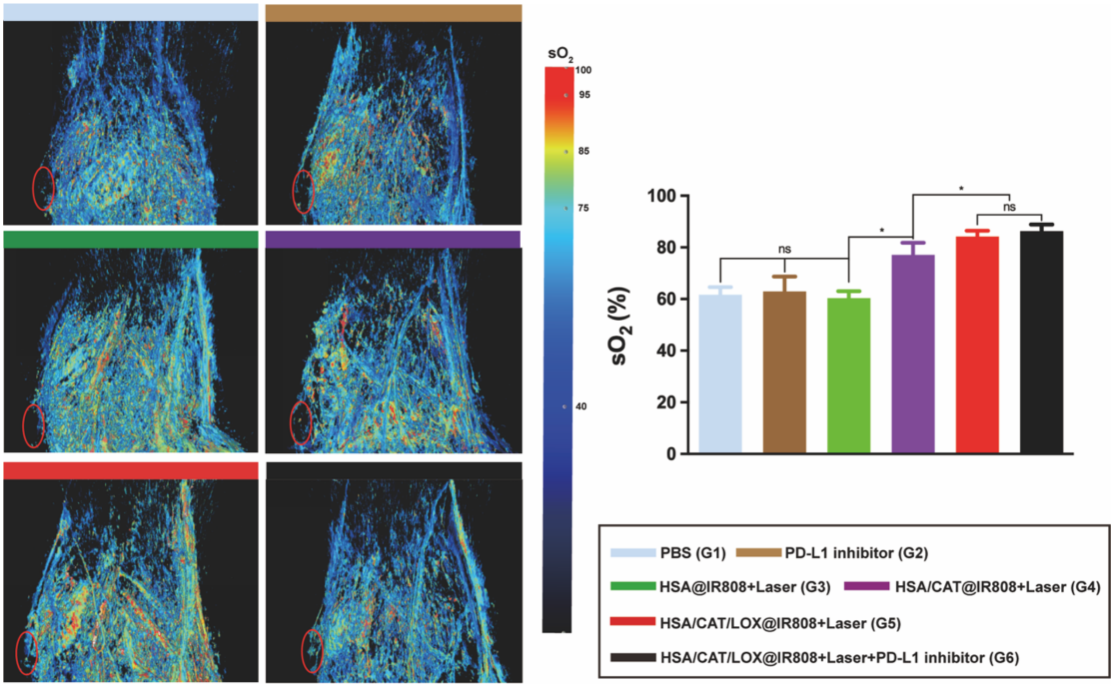

Fig. S31. Photoacoustic imaging of tumor oxygen saturation *in vivo*. The tumor tissues (outlined in red) were imaged *in vivo* using the LOIS-3D Plus system. The blood oxygen saturation (sO_2) levels were quantified, and the corresponding bar chart shows the results of sO_2 measurements in different treatment groups. Data are presented as mean ± SD. Statistical analyses were performed using one-way ANOVA with Tukey's post hoc tests.

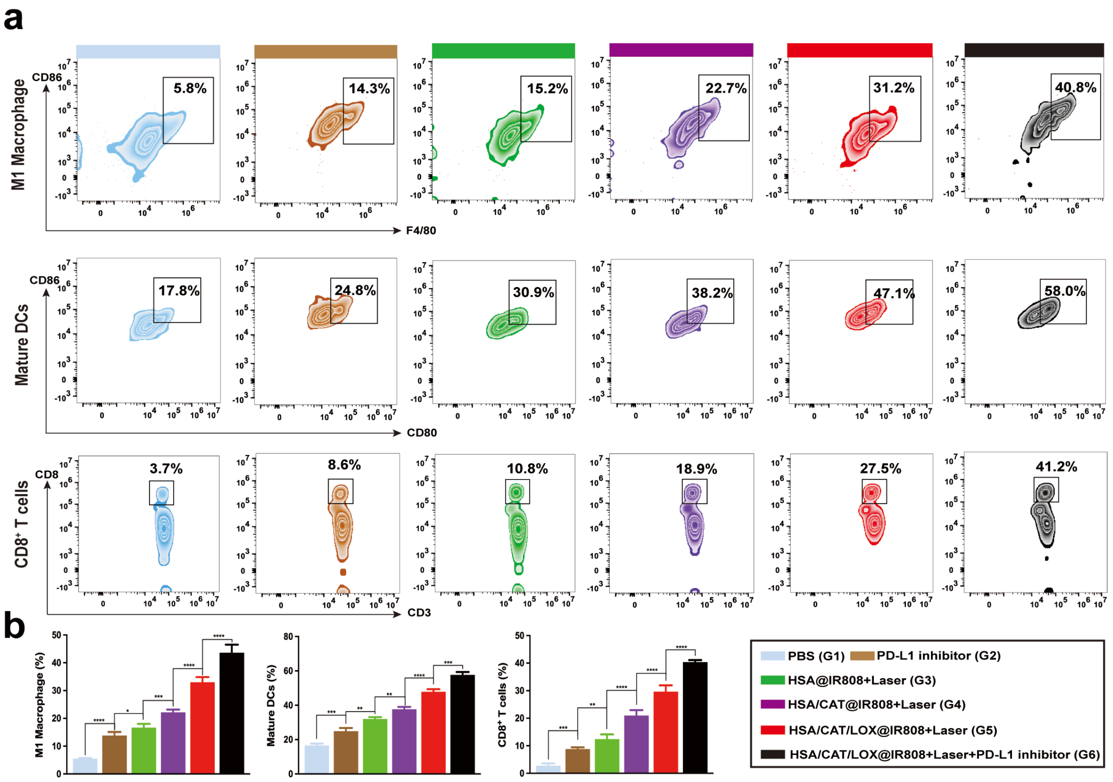

Fig. S32. Flow cytometry analysis of spleen immune cell populations. Flow cytometry results showing changes in macrophage polarization, DC maturation, and CD8$^+$ T cell frequencies in the spleen across different treatment groups, indicating enhanced immune activation following combination therapy. Data are presented as mean ± SD. Statistical analyses were performed using one-way ANOVA followed by Tukey's post hoc tests.

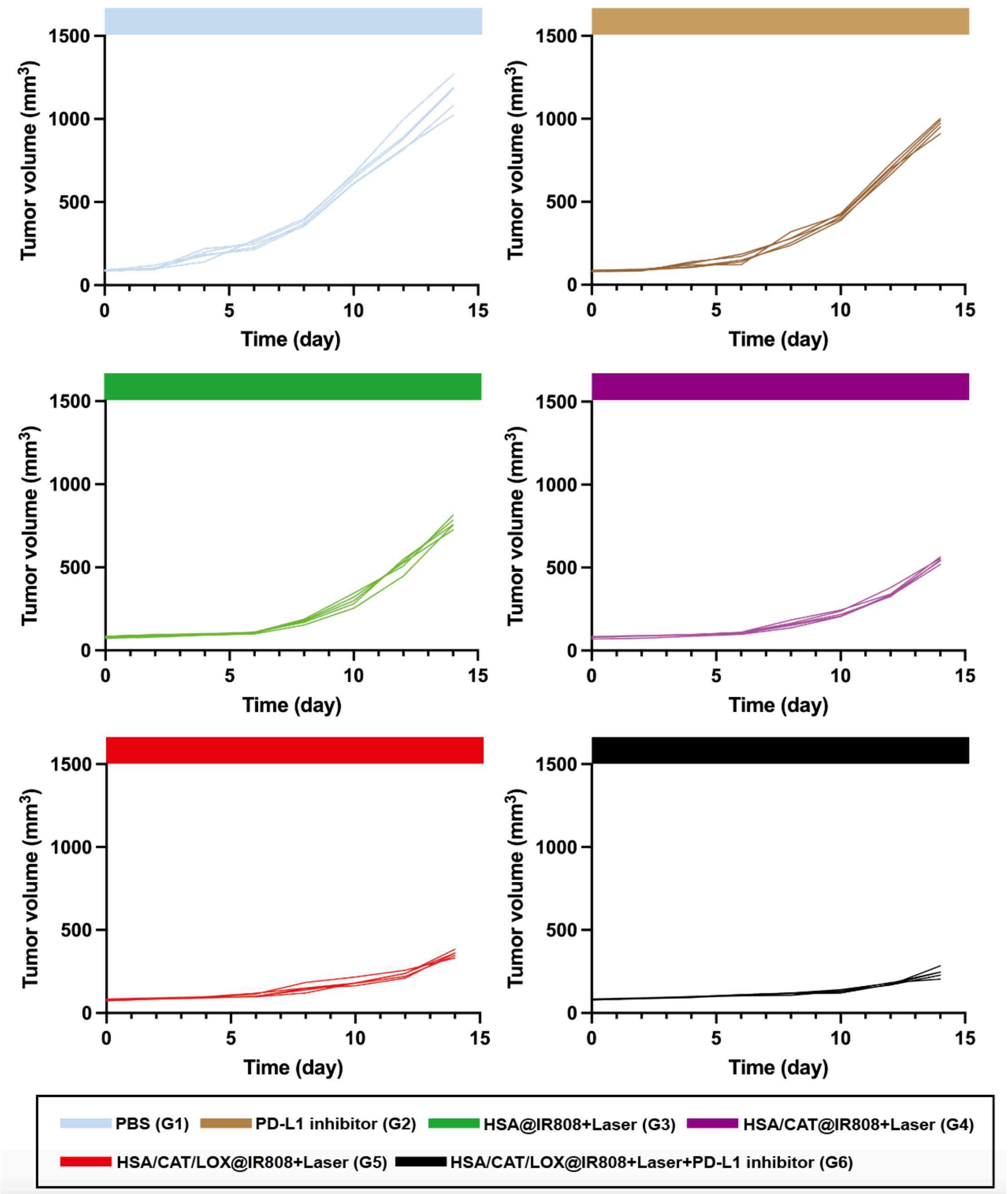

Fig. S33. Monitoring of contralateral tumor size for 14 days. Tumor growth in the contralateral site was tracked for 14 days, demonstrating varying degrees of inhibition across treatment groups, with the combination therapy (G6) showing the most significant suppression.

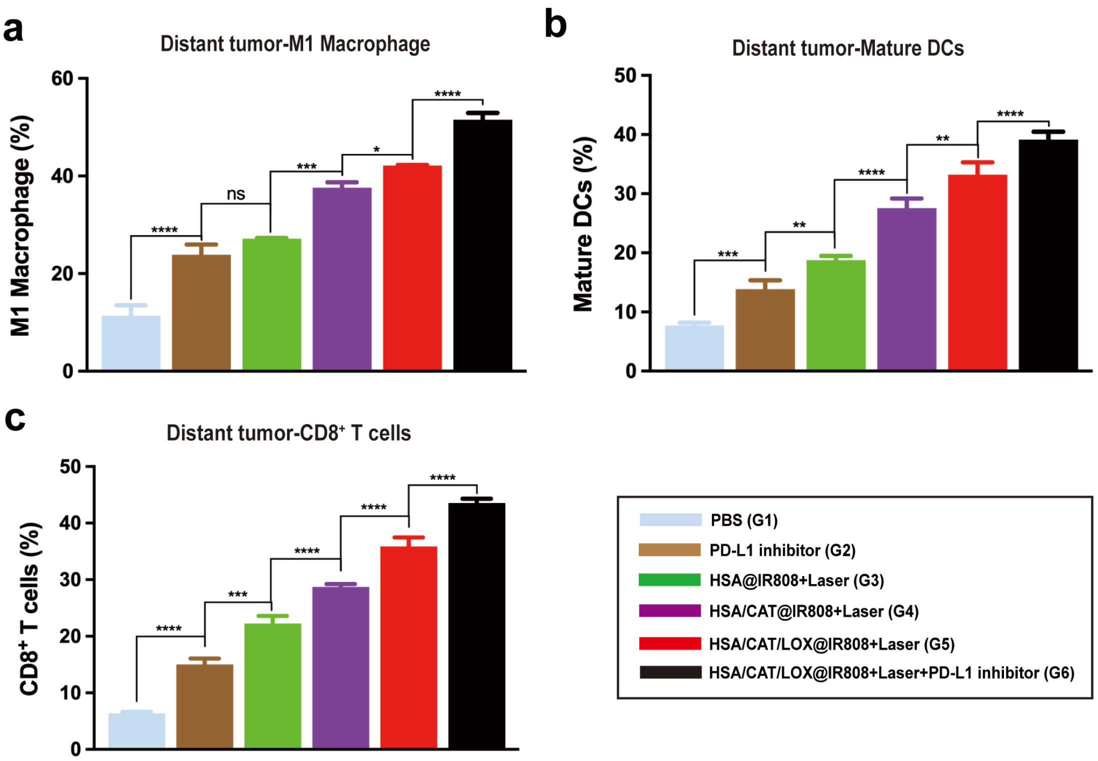

Fig. S34. Flow cytometry analysis of immune cell populations in contralateral tumors. Quantification of macrophage M1 polarization, DC maturation, and $CD8^+$ T cell activation in contralateral tumors across different groups, highlighting enhanced immune activation and tumor immune modulation following combination therapy. Data are presented as mean ± SD. Statistical analyses were performed using one-way ANOVA with Tukey's post hoc tests.

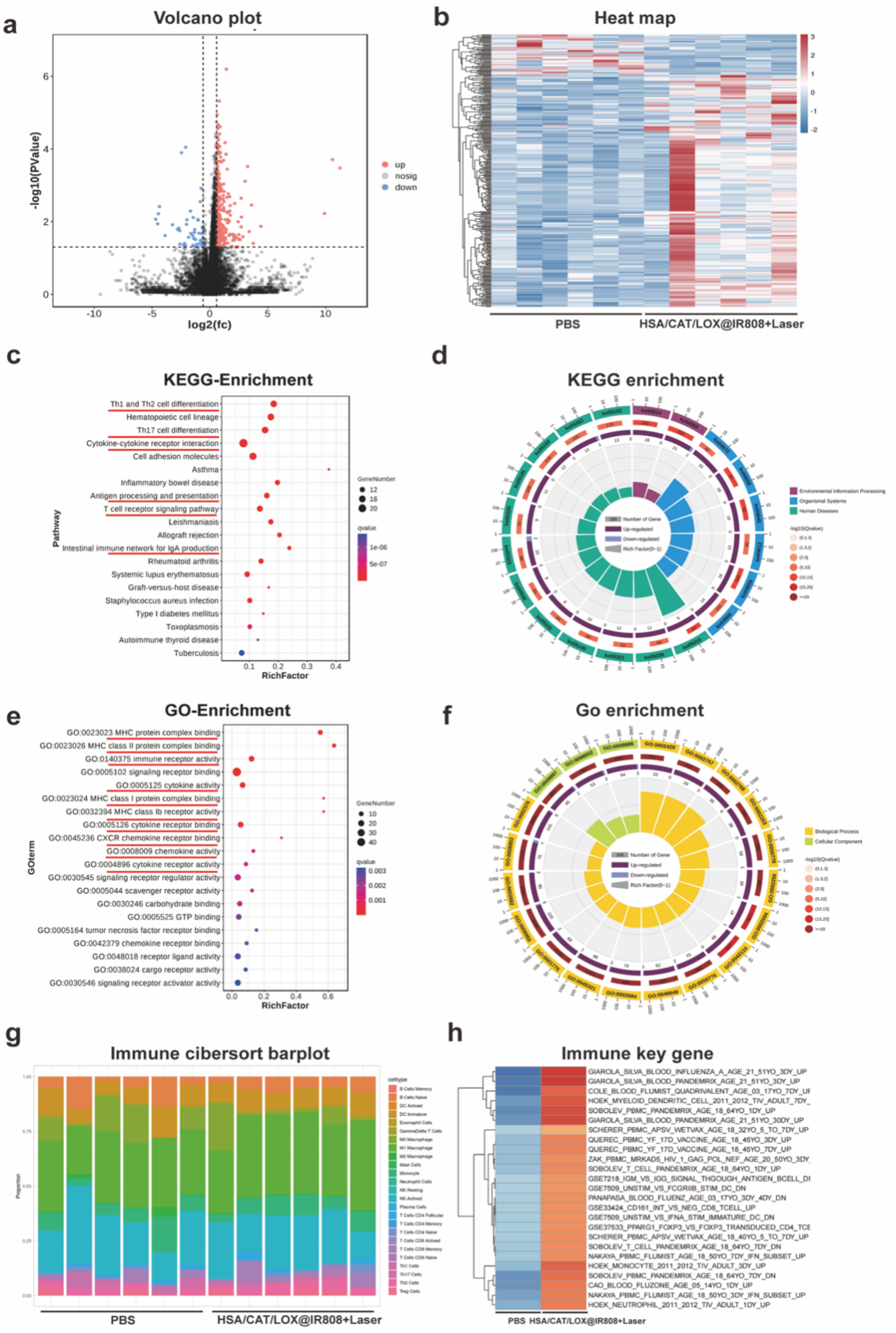

Fig. S35. Transcriptomic analysis. (a) Volcano plot illustrating differentially expressed genes between the PBS and HSA/CAT/LOX@IR808+Laser groups *in vivo*. (b) Heatmap of gene expression profiles. (c-f) KEGG and GO enrichment analyses highlighting immune-related pathway activation. (g, h) Immune infiltration analysis showing enhanced expression of immune-related gene sets.

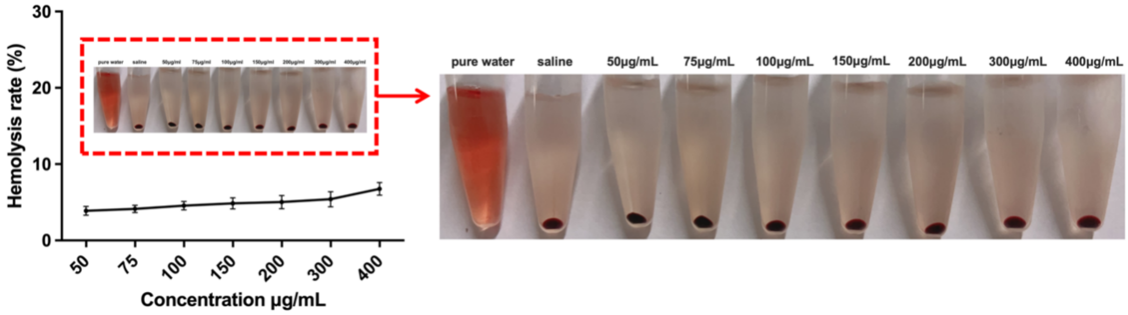

Fig. S36. The hemolysis assay revealed no hemolysis at concentrations between 50 and 400 μg/mL, suggesting that the protein vesicle do not harm red blood cells.

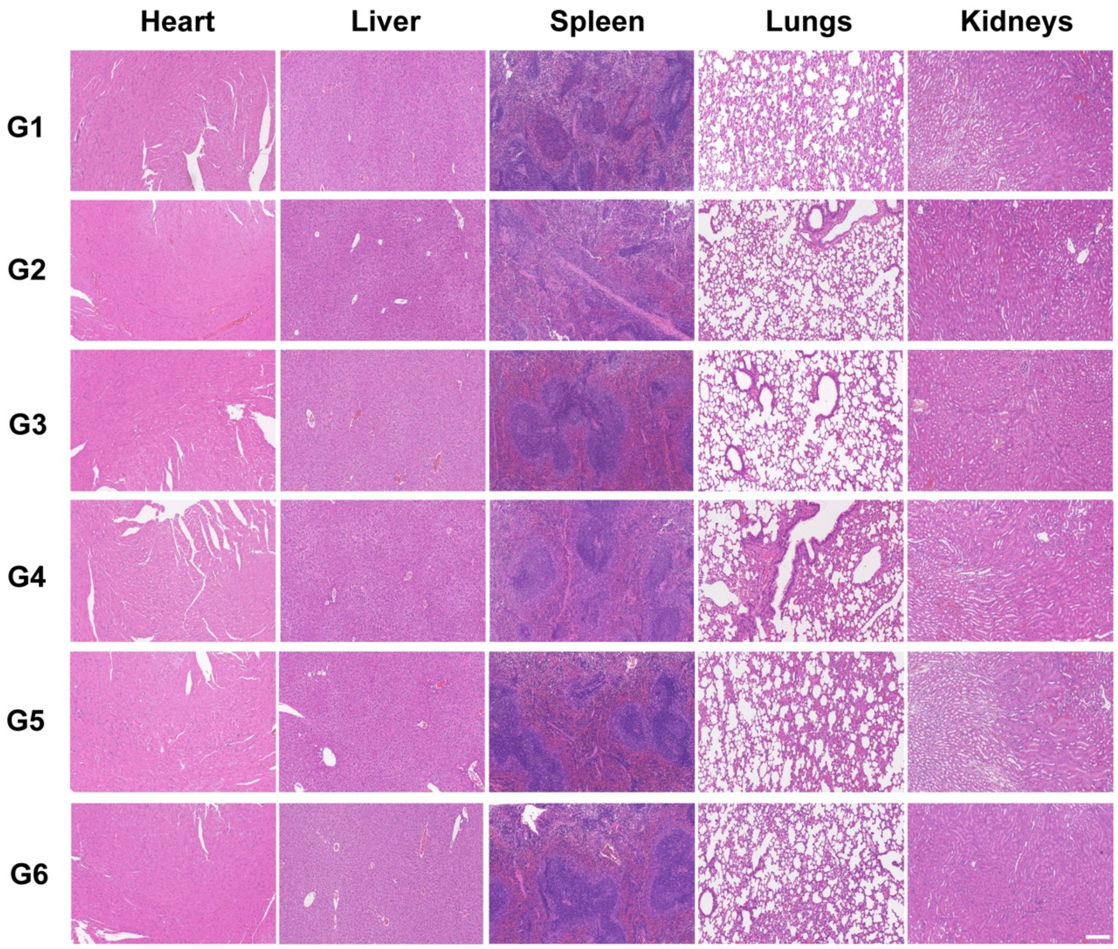

Fig. S37. Histopathological examination of major organs from all six groups of C3H/He mice showed no significant tissue damage, further validating the safety of the protein vesicle. Scale bar: 100 μm.

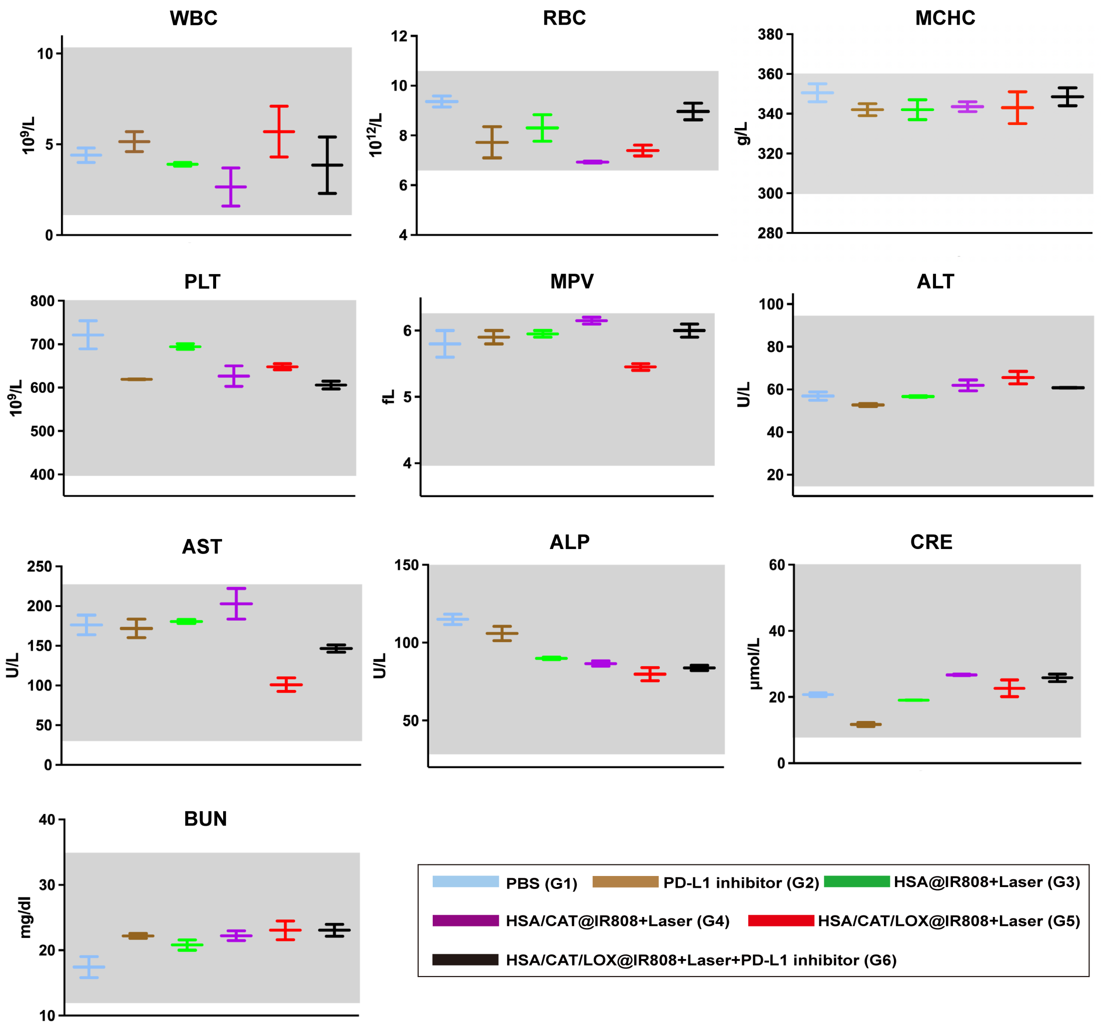

Fig. S38. Hematological parameters (WBC, RBC, MCHC, PLT, MPV) and biochemical parameters (ALT, AST, ALP, CRE, BUN) in all groups remained within normal ranges. Data are presented as mean ± SD.
